# Identification of disulfidptosis-associated genes and characterization of immune cell infiltration in thyroid carcinoma

**DOI:** 10.18632/aging.205897

**Published:** 2024-06-04

**Authors:** Siyuan Song, Jie Zhou, Li Zhang, Yuqing Sun, Qiling Zhang, Ying Tan, Xiqiao Zhou, Jiangyi Yu

**Affiliations:** 1 Department of Endocrinology, Jiangsu Province Hospital of Chinese Medicine, Affiliated Hospital of Nanjing University of Chinese Medicine, Nanjing, China; 2 Department of Endocrinology, Huaian Hospital of Huaian City, Huaian, China; 3 Department of Endocrinology, Huaian Cancer Hospital, Huaian, China

**Keywords:** disulfidptosis, thyroid carcinoma, immune cell infiltration, prognostic model

## Abstract

Objective: The primary objective of this study is to conduct a comprehensive screening and analysis of differentially expressed genes related to disulfidoptosis (DEDRGs) in thyroid carcinoma (THCA). This entails delving into the intricate characterization of immune cell infiltration within the THCA context and subsequently formulating and validating a novel prognostic model.

Method: To achieve our objectives, we first delineated two distinct subtypes of disulfidoptosis-related genes (DRGs) via consensus clustering methodology. Subsequently, employing the limma R package, we identified the DEDRGs critical for our investigation. These DEDRGs underwent meticulous validation across various databases, alongside an in-depth analysis of gene regulation. Employing functional enrichment techniques, we explored the potential molecular mechanisms underlying disulfidoptosis in THCA. Furthermore, we scrutinized the immune landscape within the two identified subtypes utilizing CIBERSORT and ESTIMATE algorithms. The construction of the prognostic model for THCA entailed intricate methodologies including univariate, multivariate Cox regression, and LASSO regression algorithms. The validity and efficacy of our prognostic model were corroborated through Kaplan-Meier survival curves and ROC curves. Additionally, a nomogram was meticulously formulated to facilitate the prediction of patient prognosis. To fortify our findings, we conducted a comprehensive Bayesian co-localization analysis coupled with rigorous *in vitro* experimentation, aimed at unequivocally establishing the validity of the identified DEDRGs.

Result: Our analyses unveiled Cluster C1, characterized by elevated expression levels of DEDRGs, as harboring a favorable prognosis accompanied by abundant immune cell infiltration. Correlation analyses underscored predominantly positive associations among the DEDRGs, further affirming their significance in THCA. Differential expression patterns of DEDRGs between tumor samples and normal tissues were evident across the GEPIA and HPA databases. Insights from the TIMER database underscored a robust correlation between DEDRGs and immune cell infiltration. KEGG analysis elucidated the enrichment of DEDRGs primarily in pivotal pathways including MAPK, PPAR signaling pathway, and Proteoglycans in cancer. Furthermore, analyses using CIBERSORT and ESTIMATE algorithms shed light on the crucial role played by DEDRGs in shaping the immune microenvironment. The prognostic model, anchored by five genes intricately associated with THCA prognosis, exhibited commendable predictive accuracy and was intricately linked to the tumor immune microenvironment. Notably, patients categorized with low-risk scores stood to potentially benefit more from immunotherapy. The validation of DEDRGs unequivocally underscores the protective role of INF2 in THCA.

Conclusion: In summary, our study delineates two discernible subtypes intricately associated with DRGs, revealing profound disparities in immune infiltration and survival prognosis within the THCA milieu. The implications of our findings extend to potential treatment strategies for THCA patients, which could entail targeted interventions directed towards DEDRGs and prognostic genes, thereby influencing disulfidptosis and the immune microenvironment. Moreover, the robust predictive capability demonstrated by our prognostic model, based on the five genes (ANGPTL7, FIRRE, ODAPH, PROKR1, SFRP5), underscores its potential clinical utility in guiding personalized therapeutic approaches for THCA patients.

## INTRODUCTION

THCA stands as the most prevalent malignancy within the realm of endocrine disorders, and its global incidence is unequivocally surging [1]. While a majority of THCA patients typically traverse a favorable prognostic trajectory, a subset grapples with formidable challenges such as distant metastasis, recurrent episodes, and suboptimal responses to therapeutic interventions, significantly impinging upon both their quality of life and survival rates [2]. Hence, the imperative quest for novel prognostic markers and therapeutic targets looms large, poised to revolutionize the landscape of THCA management and augment the well-being of affected individuals.

Disulfidptosis, emerging as a distinctive paradigm of cellular demise, operates autonomously from established programmed death pathways encompassing apoptosis, ferroptosis, necroptosis, and copper-induced death [3]. It delineates a rapid cellular demise incited by the perturbation of disulfide equilibrium within cellular confines. Remarkably, amidst glucose-deprived milieus, an anomalous accrual of disulfides, notably cystine, ensues within cells boasting heightened expression of SLC7A11. This accrual precipitates a state of disulfide stress, instigating a surge in disulfide bond densities within the actin-regulated cytoskeleton. Consequently, pronounced cytoskeletal contraction and detachment from the cellular membrane ensue, engendering disruptive distortions in cytoskeletal architecture, culminating inexorably in cellular demise. Ongoing investigations into disulfidptosis predominantly orbit around cancerous cell cohorts exhibiting augmented SLC7A11 expression amidst glucose-deprived microenvironments [4, 5]. Studies have discerned compelling associations linking disulfidptosis with a panoply of malignancies, spanning from bladder cancer [6] to hepatocellular carcinoma [7] and lung adenocarcinoma [8]. Nevertheless, the enigmatic nexus between disulfidptosis, as an incipient mechanism of cellular demise, and THCA remains shrouded in ambiguity, awaiting comprehensive elucidation.

**Figure 1:**
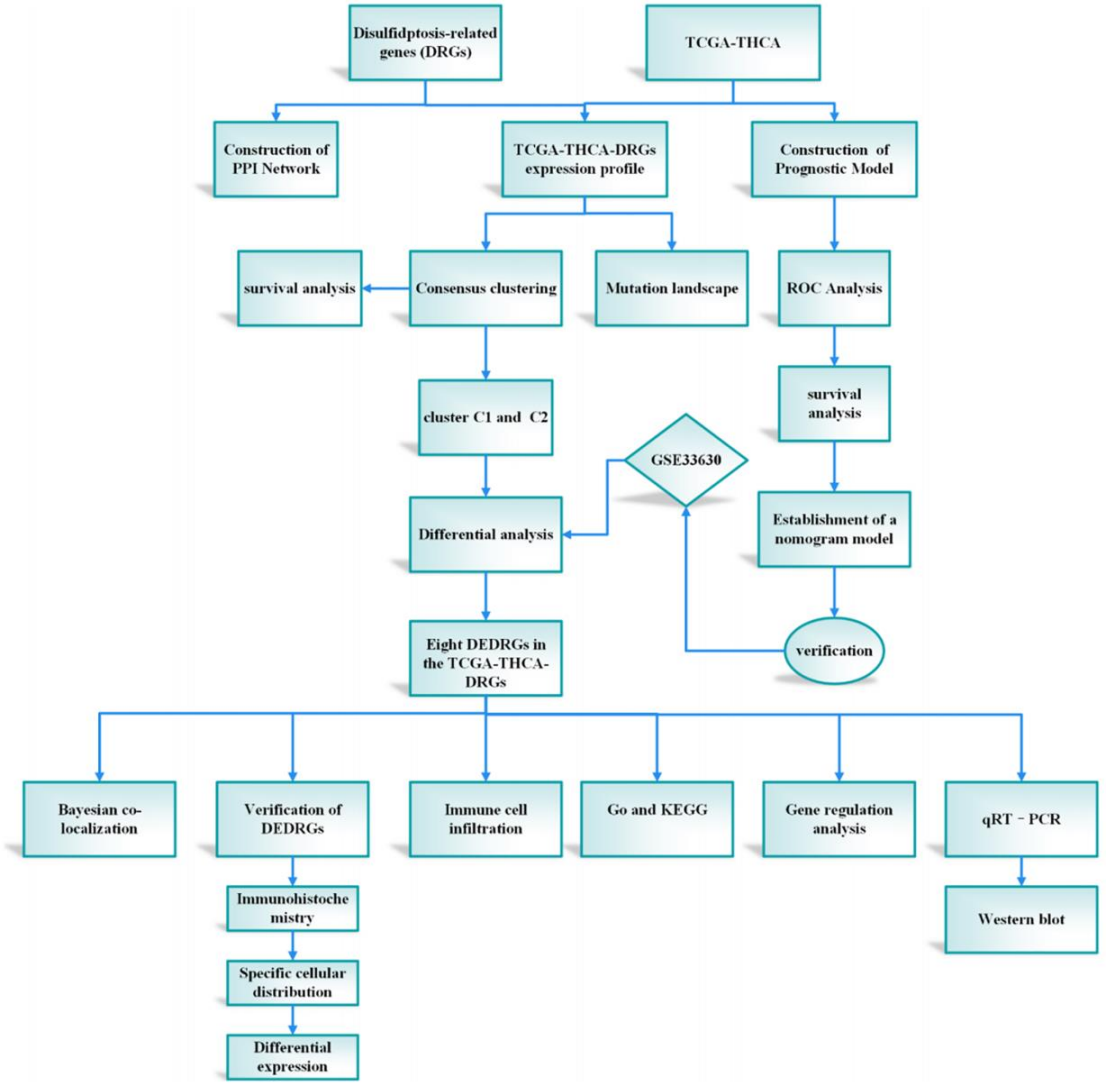
The protocol of our study procedure.

The integration of machine learning methodologies has emerged as a cornerstone in the realm of gene discovery, particularly in discerning genes harboring diagnostic prowess. Harnessing the power of machine learning algorithms has heralded a paradigm shift, markedly amplifying the precision in discerning differentially expressed genes (DEGs) within microarray datasets [9]. In the context of our investigation, we embarked upon the construction of a prognostic framework predicated upon the intricate interplay among the attributes characterizing DRGs. This prognostic model stands poised as a harbinger of elucidating hitherto uncharted biological cascades underpinning the genesis and progression of THCA, thereby unfurling vistas for the identification of novel diagnostic modalities, prognostic markers, and therapeutic targets of paramount clinical significance. The meticulously delineated study protocol is visually encapsulated in Figure 1.

## METHODS

### Bioinformatics analysis

#### Source and processing of data

The genomic sequencing data for THCA were obtained from the Cancer Genome Atlas (TCGA) database (https://portal.gdc.cancer.gov/). This study employed expression data from TCGA-THCA-FPKM, alongside pertinent clinical information from 507 THCA cases, encompassing demographics such as age, gender, stage, TNM classification, survival status, and duration. The characteristics of the THCA patients within the TCGA database are outlined in Supplementary Table 1. Before analyzing the expression profile data, transcripts per million (TPM) from the exon model per thousand bases underwent standardization. Drawing from prior literature [3, 4, 10, 11], a curated set of 106 DRGs was compiled and detailed in Supplementary Table 2.

Additionally, the THCA dataset GSE33630, comprising 49 THCA samples and 45 normal tissues, was accessed from the GEO database (https://www.ncbi.nlm.nih.gov/geo/). Leveraging the labeling information of the GPL570 platform, probes were converted into corresponding gene symbols. This dataset served as the validation cohort for our study.

#### Consensus clustering

Consensus clustering serves as a methodological cornerstone for discerning the optimal number of clusters across diverse clustering algorithms [12]. In our study, leveraging the expression profiles of DRGs, we employed the ConsensusClusterPlus R package to effectively stratify THCA samples into discernible subtypes. To ensure robustness, the ConsensusClusterPlus command was iterated 1000 times, thereby fortifying classification stability. The determination of the cluster number (k) was facilitated by the cumulative distribution function (CDF) of uniform clustering. Subsequently, survival analysis was meticulously conducted, juxtaposing Cluster C1 and Cluster C2 based on survival status and duration, utilizing the survival R package. Moreover, we undertook a comprehensive assessment of survival disparities concerning gender, disease stage, and TNM classification.

#### Differential analysis

The expression levels of DRGs were extracted from the TCGA-THCA dataset utilizing the limma package. Subsequently, the transcriptomic data of TCGA-THCA-DRGs underwent rigorous analysis employing the DESeq2 R package. Employing stringent criteria, wherein |log2FC| > 1.5 and a corrected *P* < 0.05 were utilized as screening thresholds, the DEDRGs were meticulously identified. Simultaneously, employing the same rigorous methodology, we endeavored to identify DEDRGs within the validation cohort. This comprehensive approach ensured consistency and reliability across datasets, facilitating robust comparative analyses.

#### Verification of DEDRGs in the different database

We employed the “Expression Boxplot” module within the GEPIA database (http://gepia.cancer-pku.cn/) [13] to evaluate the differential expression patterns of DEDRGs between normal tissues and THCA samples. This facilitated a comprehensive assessment of the transcriptional alterations of DEDRGs across distinct biological contexts. Additionally, we utilized the “Survival Plots” module within the same platform to delve into the prognostic implications associated with DEDRGs. This enabled a detailed exploration of the potential prognostic significance of DEDRGs in the context of THCA patient outcomes. Furthermore, to elucidate the precise cellular distribution and immunohistochemical (IHC) characteristics of DEDRGs, we conducted verification using the Human Protein Atlas (HPA) database (https://www.proteinatlas.org/) [14]. This comprehensive analysis provided valuable insights into the spatial localization and potential functional roles of DEDRGs within cellular frameworks. Moreover, the TIMER database (https://cistrome.shinyapps.io/timer/) [15] served as an essential resource for examining the intricate relationship between DEDRGs and immune cell infiltration. This facilitated a thorough interrogation of the immune microenvironment within the context of THCA, shedding light on potential immunomodulatory roles of DEDRGs [14].

#### Gene regulation analysis of DEDRGs

GenDoma represents a pioneering effort in China, constituting the premier comprehensive database encompassing human genes and diseases. By furnishing a multi-faceted analysis, GenDoma is poised to significantly bolster the realm of precision medicine. A central tenet of this study revolves around the Gene Regulatory Network (GRN), which serves as a compendium of molecular regulators governing gene expression at both mRNA and protein levels. In the pursuit of elucidating the regulatory landscape of DEDRGs, we harnessed the resources of the GenDoma. Through this platform, we accessed the GRN pertaining to DEDRGs, thereby unraveling the intricate web of regulatory interactions governing their expression dynamics.

#### GO and KEGG analysis

Utilizing the clusterProfiler R package [16], we conducted an in-depth analysis to elucidate the potential biological functions [17, 18] of the DEDRGs. Concurrently, we endeavored to explore their underlying molecular mechanisms. A significance threshold of *P* < 0.05 was applied to discern statistically significant findings. To present the results in a visually intuitive manner, we employed a diverse array of visualization techniques, including bubble charts, circle charts, and lollipop charts. These visualizations were meticulously crafted using the ggplot2 R package, facilitating the elucidation and interpretation of complex biological phenomena encoded within the DEDRGs.

#### Analysis of mutation landscape

Somatic mutation data pertinent to THCA were meticulously retrieved from the esteemed TCGA database in the form of Mutation Annotation Format (MAF) files. Subsequently, to offer a comprehensive visualization of the mutational landscape, a Waterfall Map was meticulously generated leveraging the Maftools R Package.

#### Correlation analysis of immune cell infiltration

In order to infer the abundance of 22 invasive immune cell types within THCA samples and normal tissues, we employed the CIBERSORT algorithm. CIBERSORT operates through a deconvolution analysis rooted in the linear support vector regression principle, thereby facilitating the prediction of the proportion of diverse cell types within gene expression profiles. A significance threshold of *P* < 0.05 was established as the standard for statistical significance [19]. The resultant data were visualized utilizing the barplot R package. Furthermore, the immune score and estimate score of THCA were meticulously computed utilizing the ESTIMATE algorithm. This allowed for a nuanced evaluation of the tumor microenvironment. To delve deeper into the correlation between DEDRGs and immune checkpoints within distinct subtypes, we employed the *Pearson* correlation coefficient to rigorously examine their association.

#### Construction and verification of prognostic model and nomogram

The glmnet R package served as a pivotal tool in conducting LASSO regression analysis, enabling the identification of pertinent variables associated with dependent variables (*P* < 0.05), while mitigating concerns related to variable collinearity [20]. This rigorous analysis facilitated the construction of a prognostic model by integrating survival time and survival status data for each sample. Subsequently, a corresponding risk score was meticulously generated, encapsulating the prognostic potential of the model. To comprehensively evaluate the predictive performance of the model, area under the curve (AUC) and receiver operating characteristic (ROC) analysis were employed, providing valuable insights into its efficacy.

Furthermore, the risk score, alongside age, gender, TNM, and stage variables, underwent rigorous assessment through univariate and multivariate Cox regression analyses. Variables exhibiting a significance level of *P* < 0.05 were identified as independent prognostic factors for THCA patient outcomes. Leveraging these independent prognostic factors, a nomogram was meticulously constructed utilizing the regplot R package. This nomogram facilitates the prediction of 1, 3, and 5-year survival rates of THCA patients, thereby offering a valuable clinical tool for prognostic assessment.

### Verification of DEDRGs

#### Bayesian co-localization analysis

Bayesian co-localization analysis plays a pivotal role in evaluating the presence of shared causal variation within two phenotypic regions. In our study, we employed the “coloc” R package to meticulously scrutinize the co-localization of seven DEDRGs with THCA, excluding FLNA due to the absence of GWAS data. This comprehensive analysis provides insights through four hypothetical posterior probabilities, elucidating the likelihood of a single variable being shared by both traits. To discern gene co-localization, we imposed a stringent criterion, stipulating that the PPH4 score exceed 0.75 [21]. The GWAS data for THCA (ebi-a-GCST90013867) were meticulously obtained from the IEU OpenGWAS platform (https://gwas.mrcieu.ac.uk/).

#### Experimental verification in vitro

##### Cell culture

The THCA cell line FTC-133 and the normal thyroid cell line Nthy-ori 3-1 were meticulously acquired from the esteemed Nanjing Kaiji Biological Company in China. These invaluable cellular resources were diligently nurtured in a nurturing environment, specifically in 1640 complete medium, meticulously supplemented with 1% penicillin-streptomycin and 10% fetal bovine serum. These cell lines were meticulously housed within a controlled cell incubator, meticulously set at a constant temperature of 37°C with 5% CO2 to ensure optimal growth conditions. Upon attaining a confluent state, typically ranging between 70% to 80%, the cells were meticulously segregated into two distinct groups: the normal group represented by FTC-133 and the control group represented by Nthy-ori 3-1. This meticulous stratification laid the groundwork for subsequent experimentation, ensuring the precise delineation of experimental parameters and the attainment of rigorous scientific insights.

##### Quantitative real-time polymerase chain reaction (qRT–PCR)

Following the compelling outcomes derived from Bayesian co-localization analysis, PDLIM1 and INF2 emerged as prime candidates for qRT–PCR validation. To initiate this crucial validation process, total RNA was meticulously extracted from both the thyroid cancer cell line FTC-133 and the normal thyroid cell line Nthy-ori 3-1. Subsequently, cDNA synthesis was meticulously performed through reverse transcription, adhering meticulously to the prescribed protocols provided by the reverse transcription kit. The qRT-PCR reaction system was then meticulously established, meticulously employing the pertinent target gene primers and PCR Master Mix. Within this meticulously curated system, GAPDH served as the internal reference, ensuring the precision and reliability of the mRNA expression analysis of PDLIM1 and INF2. Employing a qRT-PCR kit, the mRNA expression levels of PDLIM1 and INF2 were meticulously assessed. PCR served as the method of choice to detect and quantify the expression levels of each gene relative to the internal reference, utilizing the well-established 2^-ΔΔCt^ method. This pivotal experiment was meticulously repeated three times to ensure robustness and reproducibility of the findings, with outcomes meticulously averaged for precise interpretation. Detailed primer sequences utilized in this experiment can be found in Supplementary Table 3.

##### Western blot

The process of protein extraction involves meticulously applying a protein lysis solution to the cell lysate, a procedure meticulously conducted in an ice bath to ensure the preservation of protein integrity. Subsequently, the total protein concentration is quantitatively determined using the BCA method, thereby ensuring the accurate assessment of protein levels. For protein analysis, the protein samples undergo separation through either 10% or 12% SDS-PAGE electrophoresis, tailored to specific experimental requirements. Following electrophoresis, the proteins are carefully transferred onto a PVDF membrane, a step crucial for facilitating subsequent immunoblotting procedures. The PVDF membrane is then immersed in a shaking solution containing 5% skimmed milk powder, effectively blocking non-specific binding sites and enhancing signal specificity.

Following the blocking step, the membrane is probed with a protein-specific primary antibody, ensuring the specific detection of the target protein of interest. Incubation with the primary antibody is carried out at 4°C for an extended duration, typically lasting 13 hours, to facilitate optimal antibody binding. Subsequently, the membrane undergoes additional incubation with the corresponding secondary antibody, typically for 1.5 hours, further amplifying the signal for enhanced detection sensitivity. After the secondary antibody incubation, protein bands are visualized using an ECL luminous kit, enabling the detection of antibody-bound protein bands on the membrane. Finally, images of the protein bands are captured using an imaging system, ensuring the accurate documentation of experimental results. Subsequent analysis and quantification of protein band intensities are conducted using ImageJ software, facilitating data interpretation and comparison across experimental conditions.

### Statistical analysis

Statistical analyses were meticulously conducted utilizing R (version 4.1.2) and GraphPad Prism (version 8.0.1) software packages. Intergroup comparisons were meticulously performed using one-way ANOVA. Systematic differences in continuous variables were meticulously assessed utilizing either independent sample *t*-tests or Mann-Whitney *U*-tests, depending on the distribution of the data. A stringent significance threshold of *P* < 0.05 was meticulously applied to determine the statistical significance of the observed results, ensuring robust and reliable interpretation of the findings.

### Availability of data and material

The datasets presented in this study can be found in online repositories. The names of the repository and accession number(s) can be found within the article.

## RESULTS

### Bioinformatics analysis

#### Landscape of DRGs

**Figure 2:**
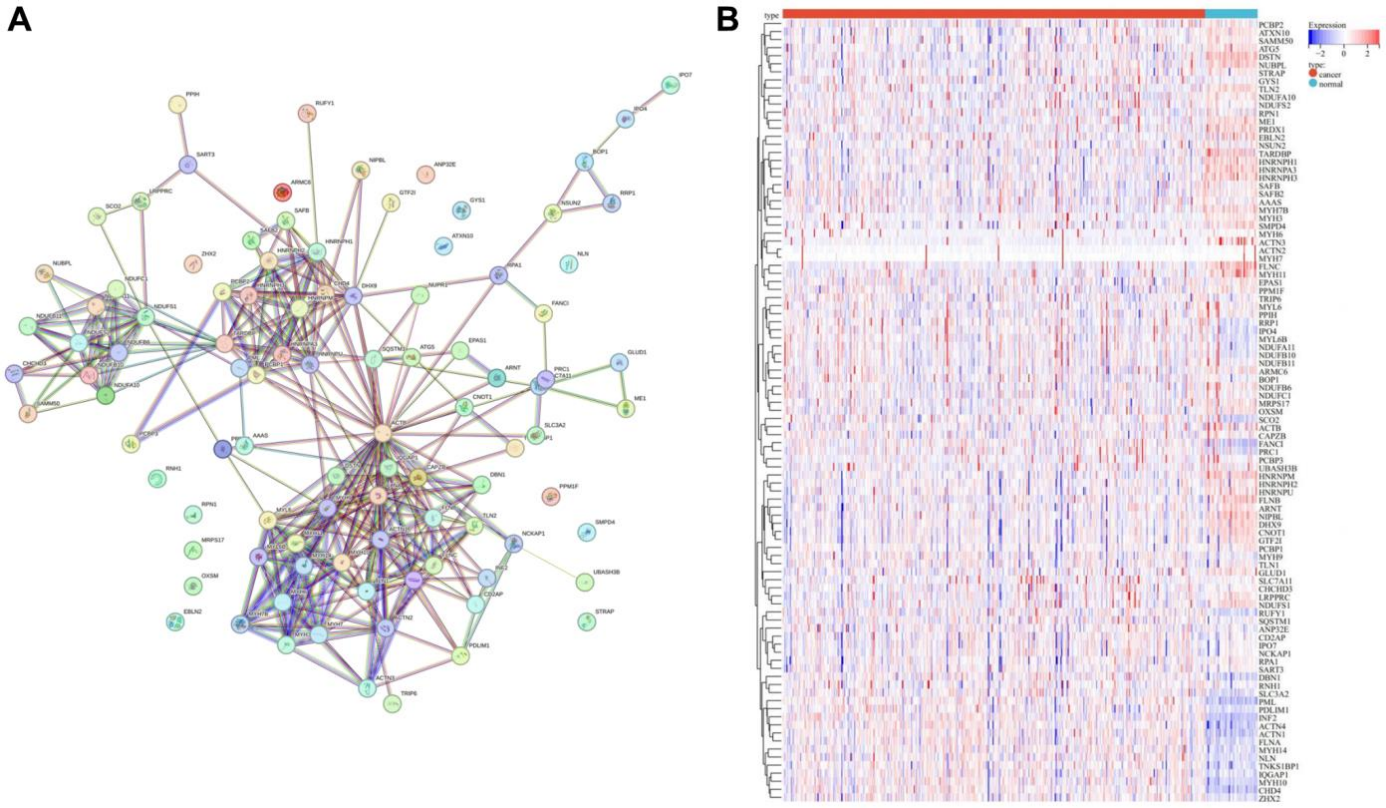
**Landscape of DRGs.** (**A**) PPI network of DRGs. (**B**) Heatmap of DRGs related expression profile.

To unravel the intricate interaction among various DRGs, we meticulously constructed a PPI network of DRGs (Figure 2A) utilizing the STRING database, a renowned resource for elucidating molecular interactions. Furthermore, the expression profiles pertinent to DRGs were meticulously visualized as a heatmap employing the pheatmap R package (Figure 2B), providing a comprehensive overview of the expression patterns across different DRGs.

#### Consensus clustering

**Figure 3:**
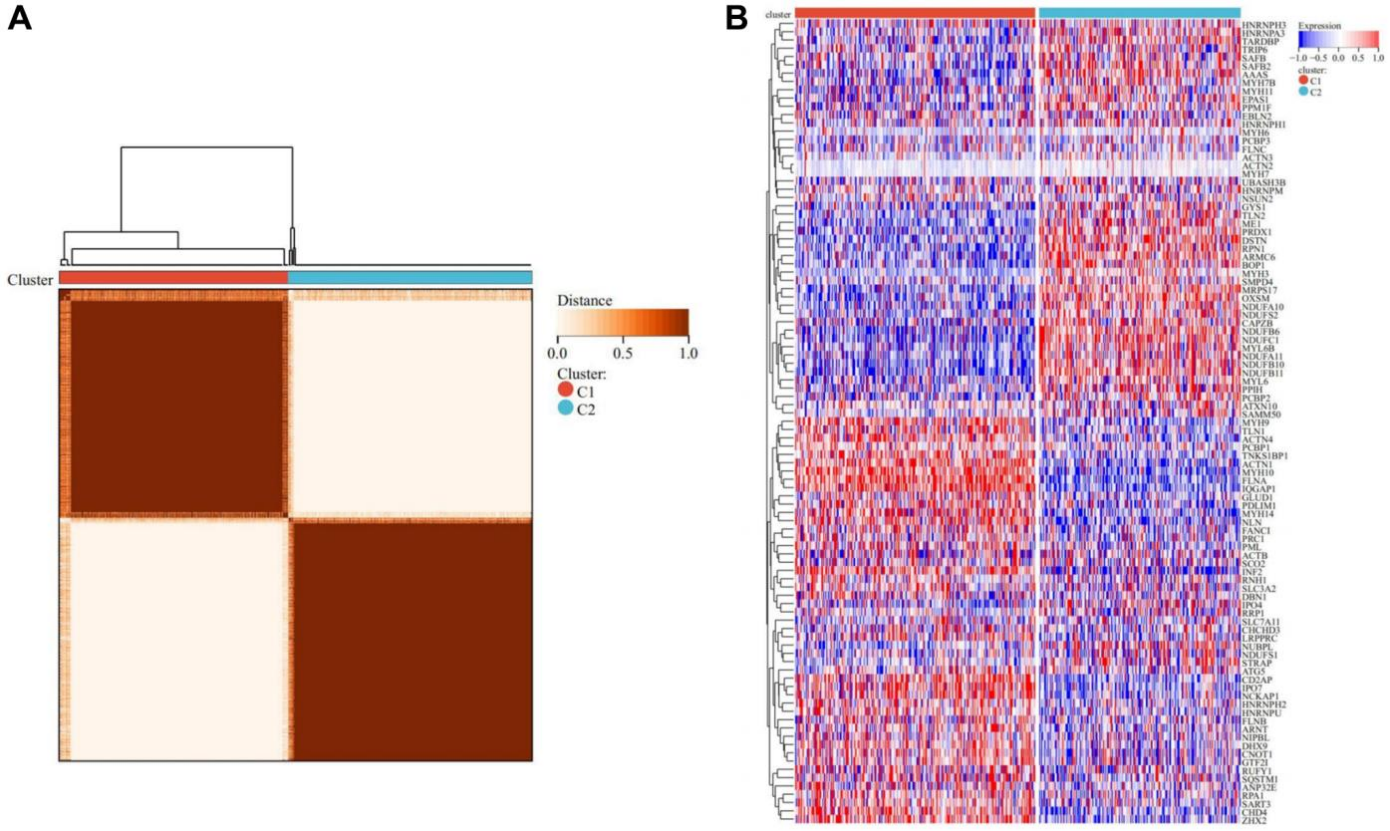
**Consensus clustering analysis.** (**A**) Represents that when k = 2, the matrix heatmap was neatly classified. (**B**) Heatmap of DRGs-related expression profile in cluster C1 and C2 subtypes.

Employing the Consensus clustering method, THCA patients were meticulously stratified into two distinct subtypes (Figure 3A, Supplementary Figure 1A–1D). The heatmap representing the two subtypes distinctly portrayed that cluster C1 exhibited a heightened expression level of DRGs, while cluster C2 showcased a comparatively lower expression level. This observation suggests a clear dichotomy, with cluster C1 corresponding to the high subtype of DRGs, and cluster C2 denoting the low subtype of DRGs (Figure 3B).

**Figure 4:**
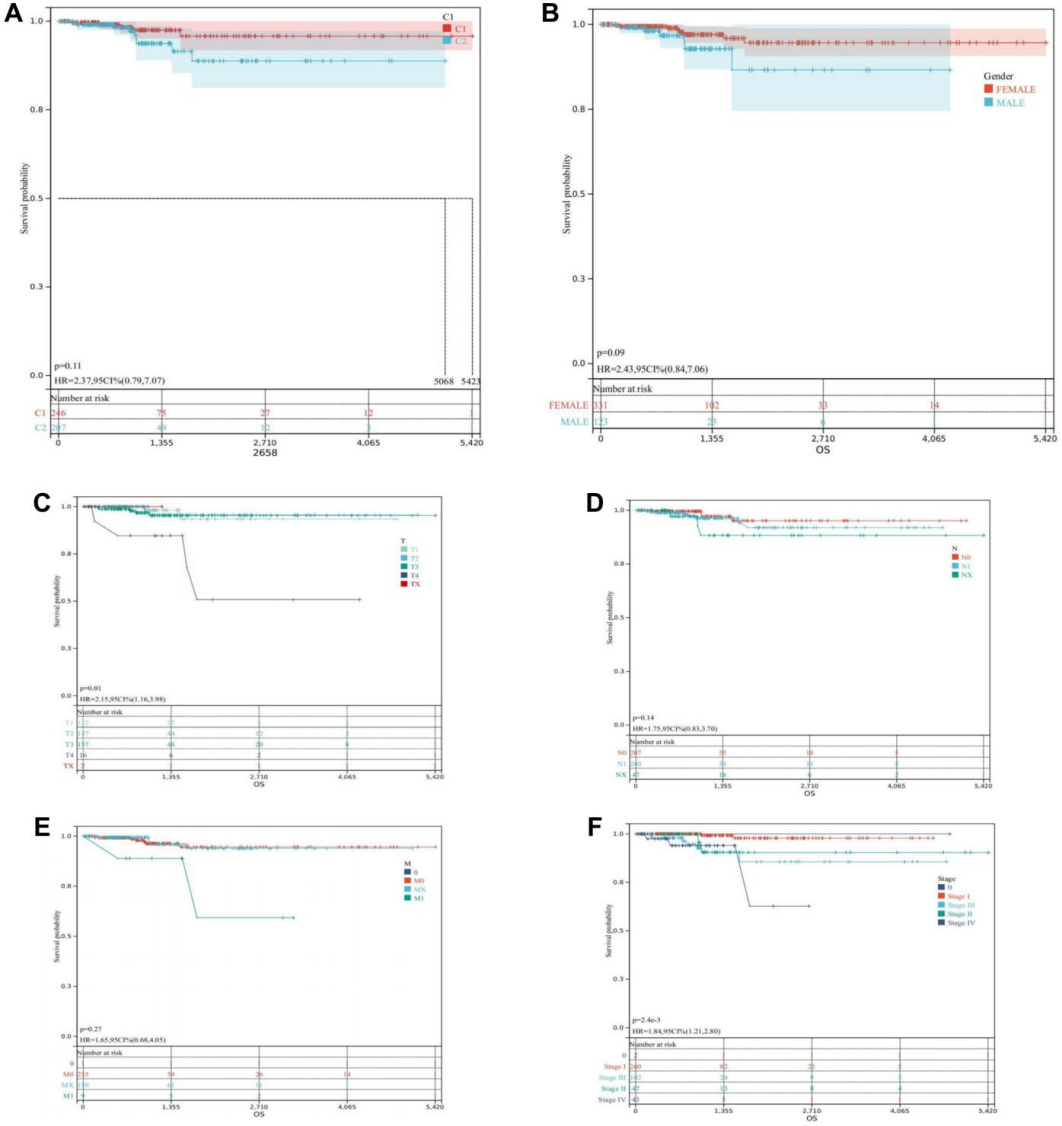
**Survival analysis in the TCGA-THCA.** (**A**) Kaplan-Meier of the two clusters. (**B**) Kaplan-Meier of THCA patients regrouped according to Gender. (**C**) Kaplan-Meier of THCA patients regrouped according to T. (**D**) Kaplan-Meier of THCA patients regrouped according to N. (**E**) Kaplan-Meier of THCA patients regrouped according to M. (**F**) Kaplan-Meier of THCA patients regrouped according to Stage.

Survival analysis unequivocally demonstrated that cluster C1, characterized by a high DRGs level, boasted a notably favorable prognosis, while cluster C2, marked by a lower DRGs level, exhibited a considerably poorer prognosis (Figure 4A). Furthermore, survival curves based on clinical characteristics elucidated that females exhibited a superior prognosis compared to males (Figure 4B), while earlier TNM staging was distinctly associated with a more favorable prognosis for THCA patients (Figure 4C–4F), underscoring the prognostic significance of clinical staging in THCA management.

#### Differential analysis

**Figure 5:**
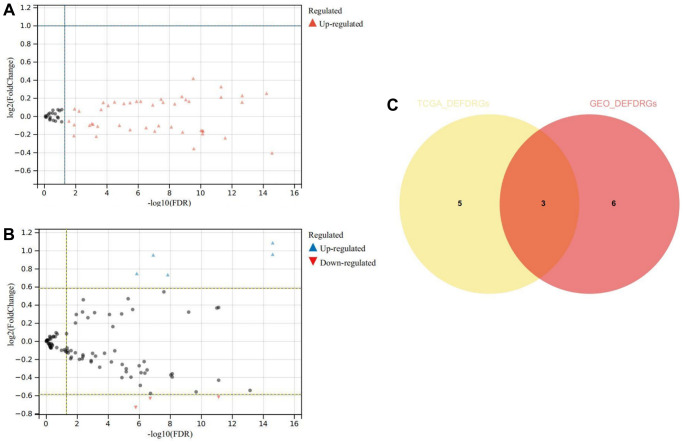
**Differential expression and correlation analysis.** (**A**) Volcanic map of DEDRGs in TCGA-THCA. (**B**) Volcanic map of DEDRGs in GSE33630. (**C**) Venn diagram of co-DEDRGs. ^*^ represents *P* < 0.05, ^***^ represents *P* < 0.001, ^****^ represents *P* < 0.0001, - represents no significant difference.

The comparative analysis of TCGA-THCA-DRGs and GSE33630-DRGs datasets meticulously revealed statistically significant differences in DRGs expression between normal and THCA samples (*P* < 0.05). From the TCGA-THCA-DRGs dataset, a total of eight DEDRGs were discerned, wherein seven genes exhibited up-regulation while one gene displayed down-regulation (Figure 5A). Notably, Cluster C1 exhibited a distinctive hallmark of heightened expression of DEDRGs, while Cluster C2 was distinctly associated with a lower expression level of DEDRGs (Supplementary Figure 2A). The correlation analysis of TCGA-DEDRGs distinctly unveiled predominantly positive correlations among the DEDRGs (Supplementary Figure 2B).

In parallel, nine DEDRGs were successfully identified from the GSE33630-DRGs dataset, comprising five up-regulated genes and four down-regulated genes (Figure 5B). Remarkably, DEDRGs exhibited a markedly elevated expression level in the THCA group compared to the normal cohort (Supplementary Figure 2C). Moreover, the correlation analysis of GEO-DEDRGs underscored predominantly positive correlations among the DEDRGs (Supplementary Figure 2D). Notably, the intersection of DEDRGs from both datasets facilitated the identification of three co-DEDRGs (Figure 5C), highlighting potential candidates with robust implications across different datasets.

#### Verification of DEDRGs in the different database

**Figure 6:**
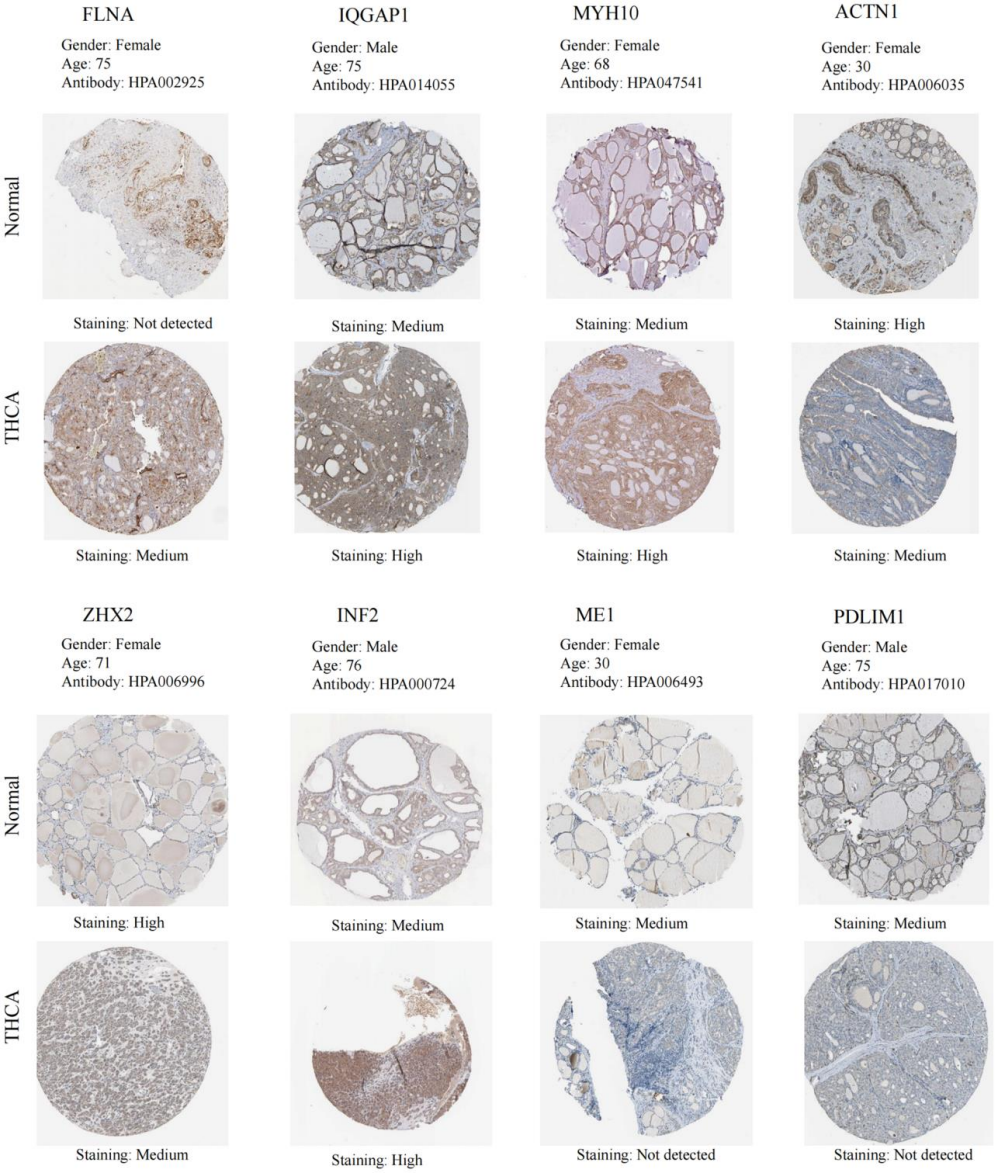
IHC of the DEDRGs in the HPA database.

The findings from the GEPIA database revealed significant differences in the expression levels of MYH10 and ME1 in THCA, with MYH10 exhibiting notably high expression and ME1 showing low expression (*P* < 0.05) (Supplementary Figure 3). Concurrently, insights gleaned from the HPA database illuminated the IHC staining patterns of the DEDRGs, showcasing distinct expression profiles between THCA samples and normal tissues (Figure 6). Specifically, MYH10 emerged as a potential risk factor for THCA, while ME1 and ZHX2 exhibited characteristics suggestive of protective factors against THCA development.

**Figure 7:**
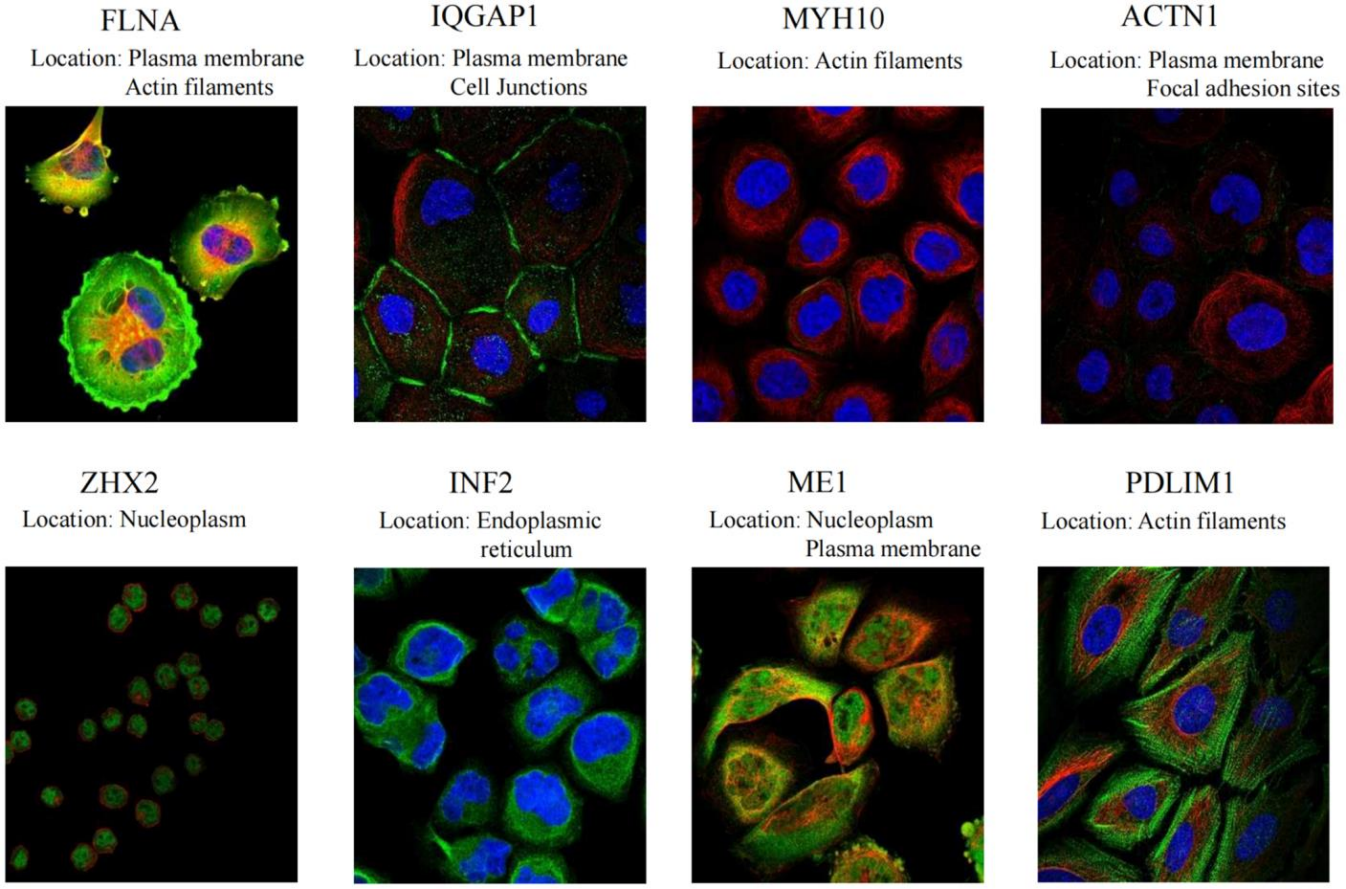
Specific spatial distribution of the DEDRGs in cells.

Moreover, Figure 7 provides a visual depiction of the specific spatial distribution of the DEDRGs within cellular contexts, unraveling deeper insights into their intracellular localization. Notably, FLNA, IQGAP1, and ACTN1 predominantly localize within the plasma membrane, hinting at their potential involvement in plasma membrane signaling and regulation. Conversely, ZHX2 and ME1 exhibit predominant localization within the nucleoplasm, suggesting roles in nuclear processes. Furthermore, MYH10 and PDLIM1 are primarily distributed within the actin filaments, indicative of their pivotal roles in actin filament signaling and receptor activation. Additionally, INF2 predominantly localizes within the endoplasmic reticulum, implicating potential roles in endoplasmic reticulum signaling. These distinctive spatial distribution patterns underscore the intricate regulatory mechanisms and diverse functional roles of the DEDRGs within THCA cells, offering valuable insights into their cellular localization and potential physiological functions.

Furthermore, analysis from the TIMER database unveiled significant correlations between DEDRGs and immune cell infiltration in THCA (Supplementary Figure 4A–4H), shedding light on the intricate interplay between DEDRGs and immune cell infiltration dynamics within the THCA microenvironment.

#### Gene regulation analysis of DEDRGs

**Figure 8:**
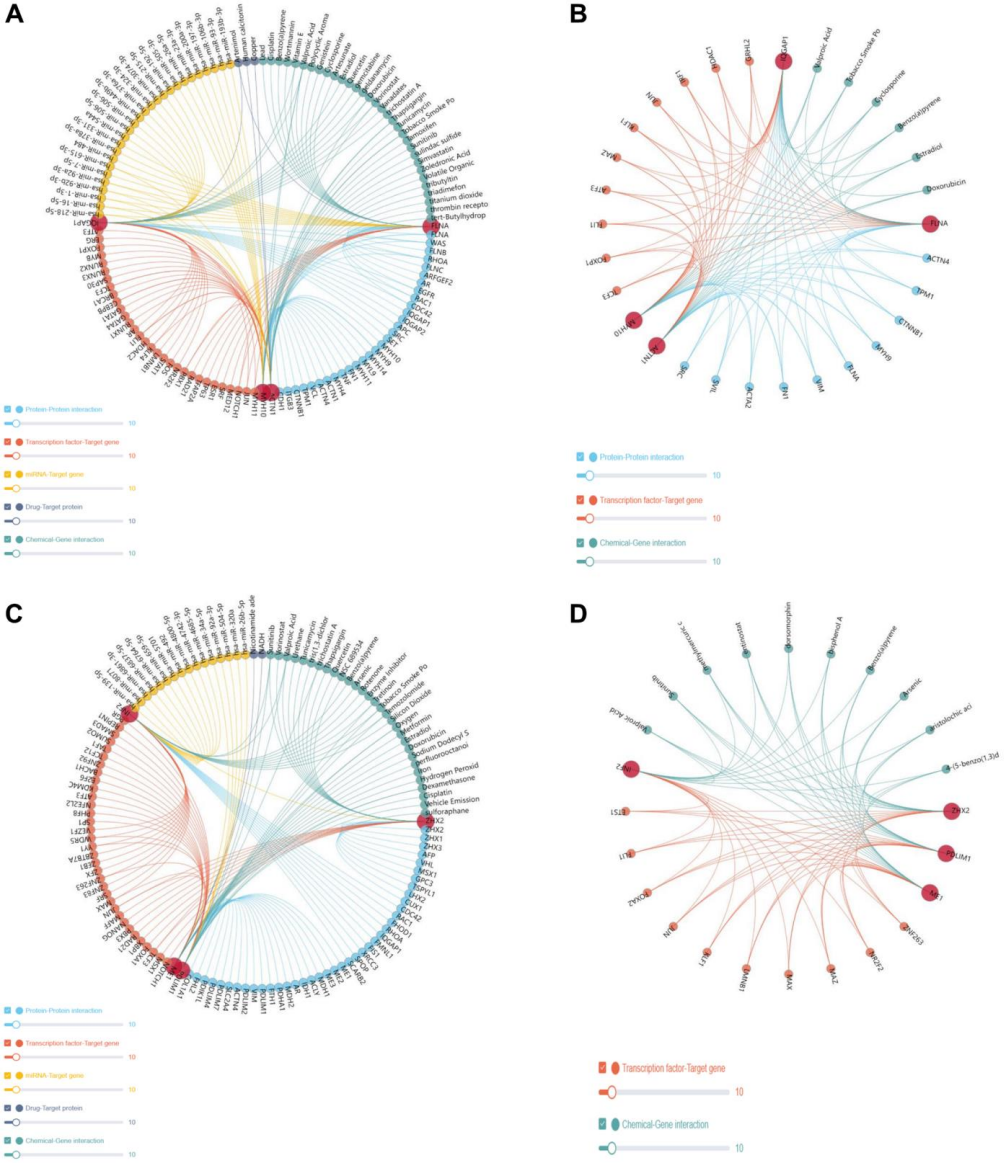
**Gene regulatory network.** (**A**, **B**) represents the gene regulatory network of the DEDRGs (FLNA, IQGAP1, MYH10, and ACTN1). (**C**, **D**) represents gene regulatory network of the DEDRGs (ZHX2, INF2, ME1, and PDLIM1).

Analysis of the gene regulation mechanisms associated with DEDRGs revealed intricate interactions with various miRNAs, transcription factors, and proteins, exerting regulatory control over the onset and advancement of THCA. Notably, the DEDRGs collectively modulate the activity of key transcription factors, including KLF1, JUN, and FLI1 (Figure 8A–8D). Furthermore, predictive modeling enabled the identification of potential targeted drugs and compounds, presenting promising prospects for future drug development and molecular therapy interventions aimed at disrupting the dysregulated pathways associated with THCA progression.

#### GO and KEGG analysis

**Figure 9:**
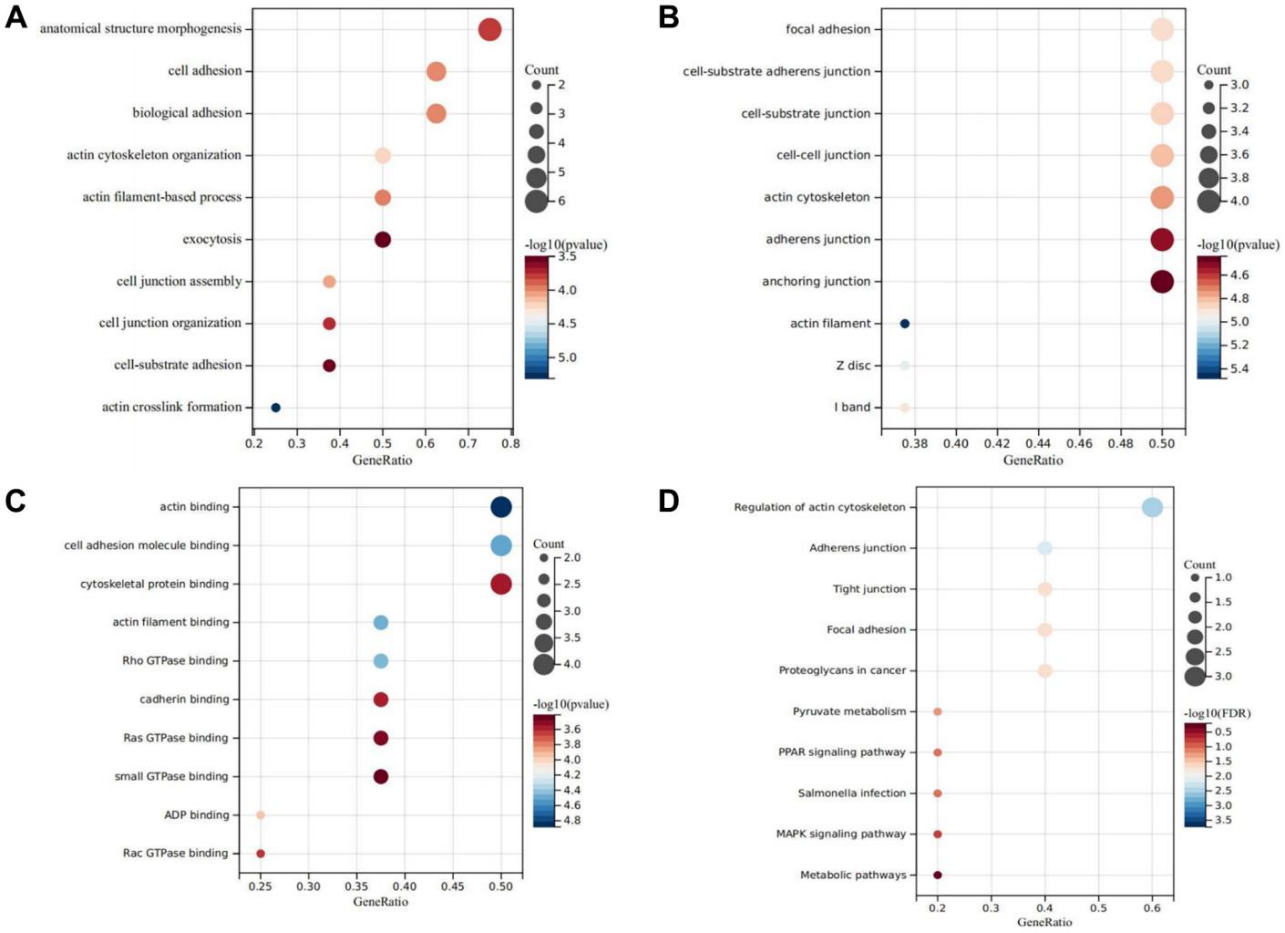
**GO and KEGG analysis.** (**A**) Represents the biological process bubble diagram of the DEDRGs in TCGA-THCA. (**B**) Represents the cellular component bubble diagram of the DEDRGs in TCGA-THCA. (**C**) Represents the molecular function bubble diagram of the DEDRGs in TCGA-THCA. (**D**) Represents the KEGG bubble diagram of the DEDRGs in TCGA-THCA. The bubble size represents the number of hub genes enrichment, and the color depth represents the level of significance.

The Gene Ontology (GO) function analysis, dissected into biological processes (BP), molecular functions (MF), and cellular components (CC) [22], unveiled significant enrichments, providing valuable insights into the functional roles of DEDRGs. Specifically, in BP, enrichment was evident in processes related to actin crosslink formation, cell junction organization, and exocytosis, highlighting their involvement in crucial cellular activities (Figure 9A). In CC, notable enrichment was observed in components associated with the actin filament and actin cytoskeleton, indicative of their pivotal roles in cytoskeletal organization and dynamics (Figure 9B). Meanwhile, MF analysis revealed significant enrichments in functions such as ADP binding, Ras GTPase binding, and actin filament binding, underscoring the diverse molecular functions of DEDRGs (Figure 9C). Furthermore, KEGG pathway analysis delineated the functional implications of DEDRGs in THCA pathogenesis. The analysis revealed significant enrichments in pathways including the MAPK, PPAR signaling pathway, and Proteoglycans in cancer, implicating their involvement in diverse signaling cascades and cellular processes critical for THCA progression (Figure 9D). These findings shed light on the intricate molecular mechanisms underlying THCA pathogenesis and highlight potential therapeutic targets for further investigation and intervention.

#### Immune cell infiltration analysis and mutation landscape

**Figure 10:**
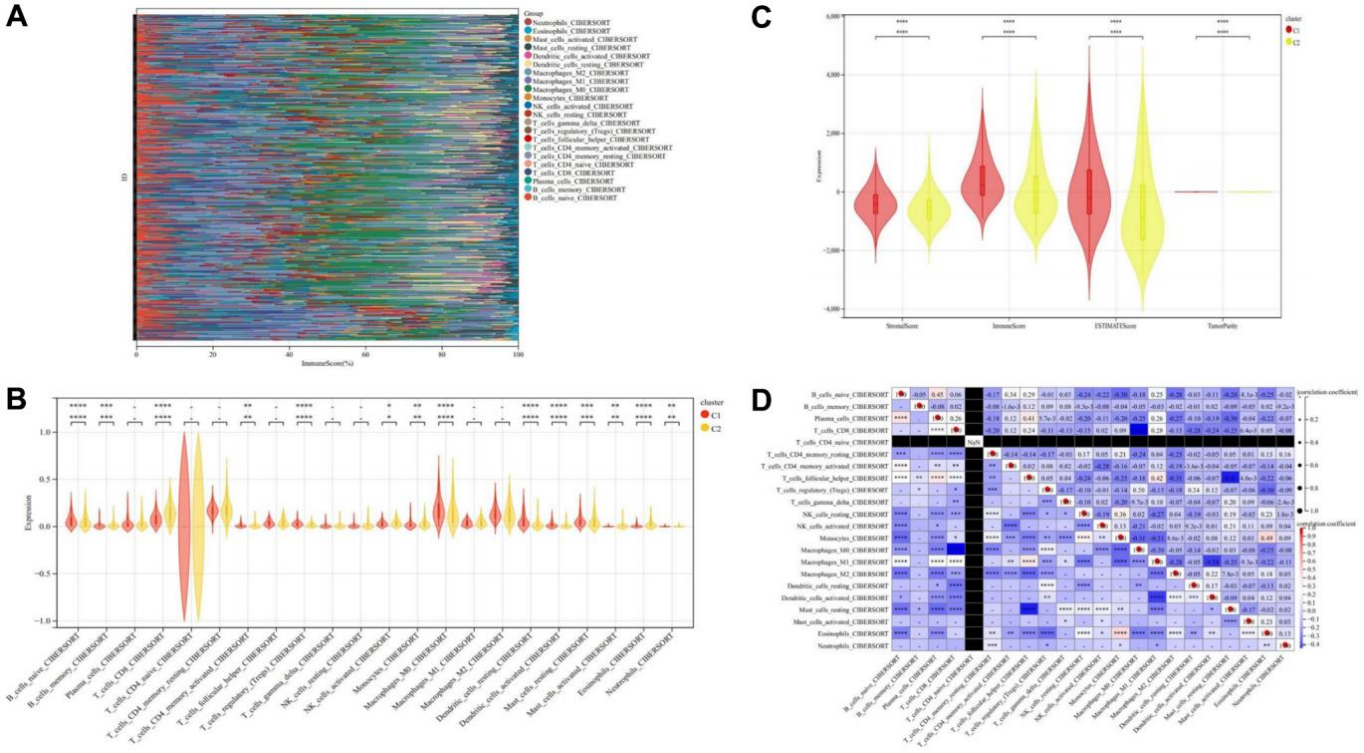
**Immune cell infiltration analysis.** (**A**) Stack diagram of immune cell infiltration. (**B**) Violin diagram of immune cell infiltration between cluster C1 and cluster C2. (**C**) Violin diagram of immune score between cluster C1 and cluster C2. (**D**) Heatmap of correlation analysis between immune cells. ^*^ represents *P* < 0.05, ^**^ represents *P* < 0.01, ^***^ represents *P* < 0.001, ^****^ represents *P* < 0.0001, - represents no significant difference.

The results of immune cell infiltration analysis revealed that Cluster C2 exhibited higher immune cell infiltration compared to Cluster C1, indicative of distinct immune microenvironments within these subtypes (Figure 10A–10C). Notably, although Cluster C2 displayed elevated immune cell infiltration, it was associated with a poorer prognosis, while Cluster C1, characterized by lower immune cell infiltration, demonstrated a better prognosis. This observation led us to postulate that Cluster C2 represents an immune rejection type, whereas Cluster C1 corresponds to an immune inflammation type, suggesting divergent immunological responses in THCA (Figure 10A–10C). Furthermore, correlation analysis of immune cells unveiled intriguing relationships among different immune cell populations (Figure 10D).

**Figure 11:**
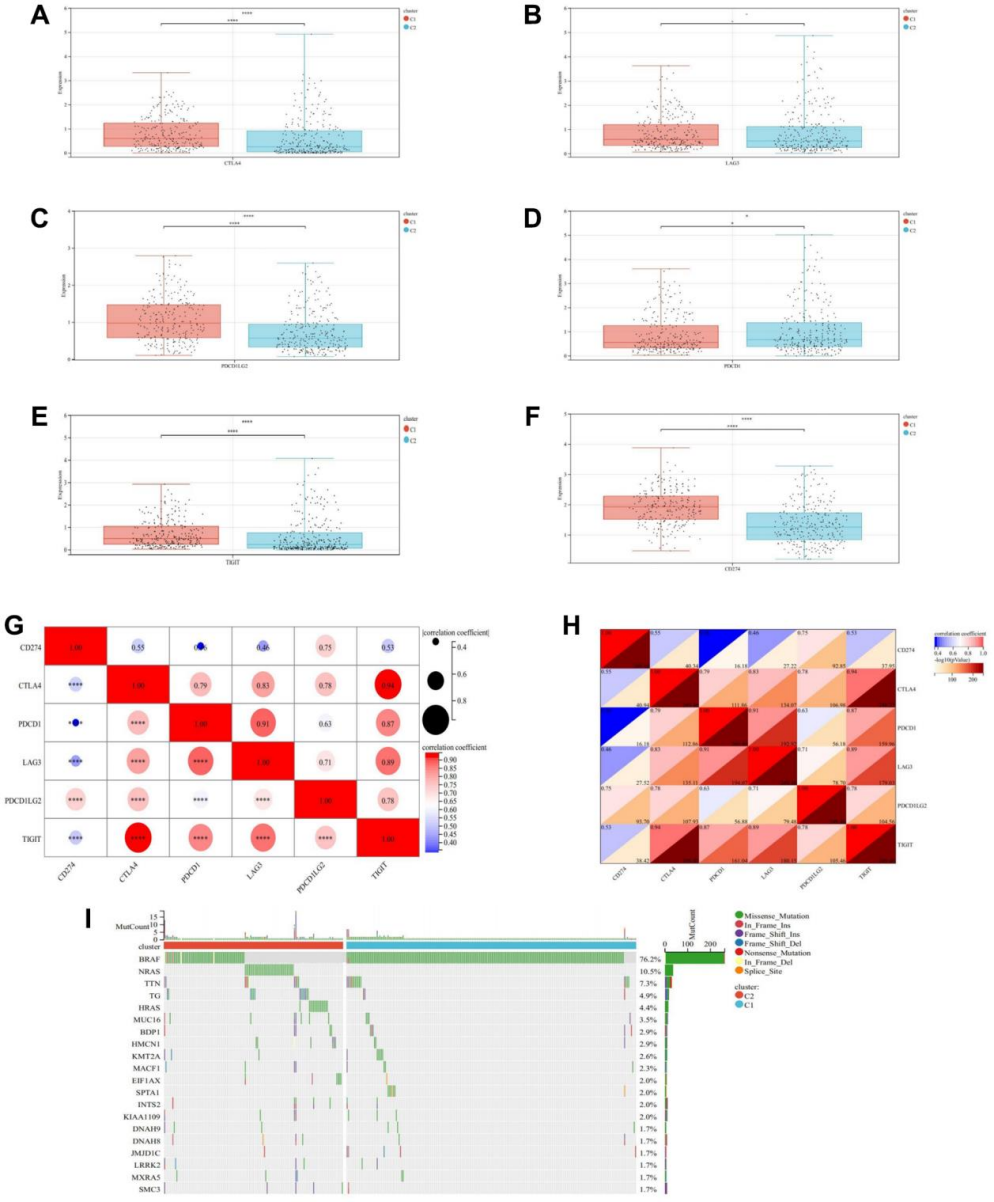
**Immune checkpoints analysis and mutation landscape.** (**A**) Expression difference of CTLA4 in two subtypes. (**B**) Expression difference of LAG3 in two subtypes. (**C**) Expression difference of PD-L1 in two subtypes. (**D**) Expression difference of PD-1 in two subtypes. (**E**) Expression difference of TIGIT in two subtypes. (**F**) Expression difference of CD274 in two subtypes. (**G**, **H**) Heatmap of correlation analysis between immune checkpoints. (**I**) Waterfall map of the Mutation landscape. ^*^ represents *P* < 0.05, ^**^ represents *P* < 0.01, ^***^ represents *P* < 0.001, ^****^ represents *P* < 0.0001, - represents no significant difference.

To further elucidate the interplay between DEDRGs and immune checkpoints in THCA, a Wilcoxon signed-rank sum test confirmed significant upregulation of TIGIT, PD-L1, CTLA4, and CD274 in Cluster C1 (*P* < 0.0001), while PD-1 was highly expressed in Cluster C2 (*P* < 0.05). Interestingly, no statistical difference in the expression of LAG3 was observed between the two subtypes, suggesting differential regulation of immune checkpoint molecules in distinct THCA subtypes (Figure 11A–11F). The correlation heatmap of immune checkpoints revealed intricate relationships among these molecules (Figure 11G, 11H).

Moreover, analysis of the mutation landscape provided insights into susceptible genes in THCA. The waterfall map of gene mutations highlighted missense mutations as the predominant classification. Remarkably, the BRAF mutation rate (76.2%) emerged as the highest in THCA, with Cluster C1 exhibiting a higher BRAF mutation frequency compared to Cluster C2, whereas NRAS mutation frequency was higher in Cluster C2 than in Cluster C1 (Figure 11I).

#### Construction and verification of prognostic model and nomogram

**Figure 12:**
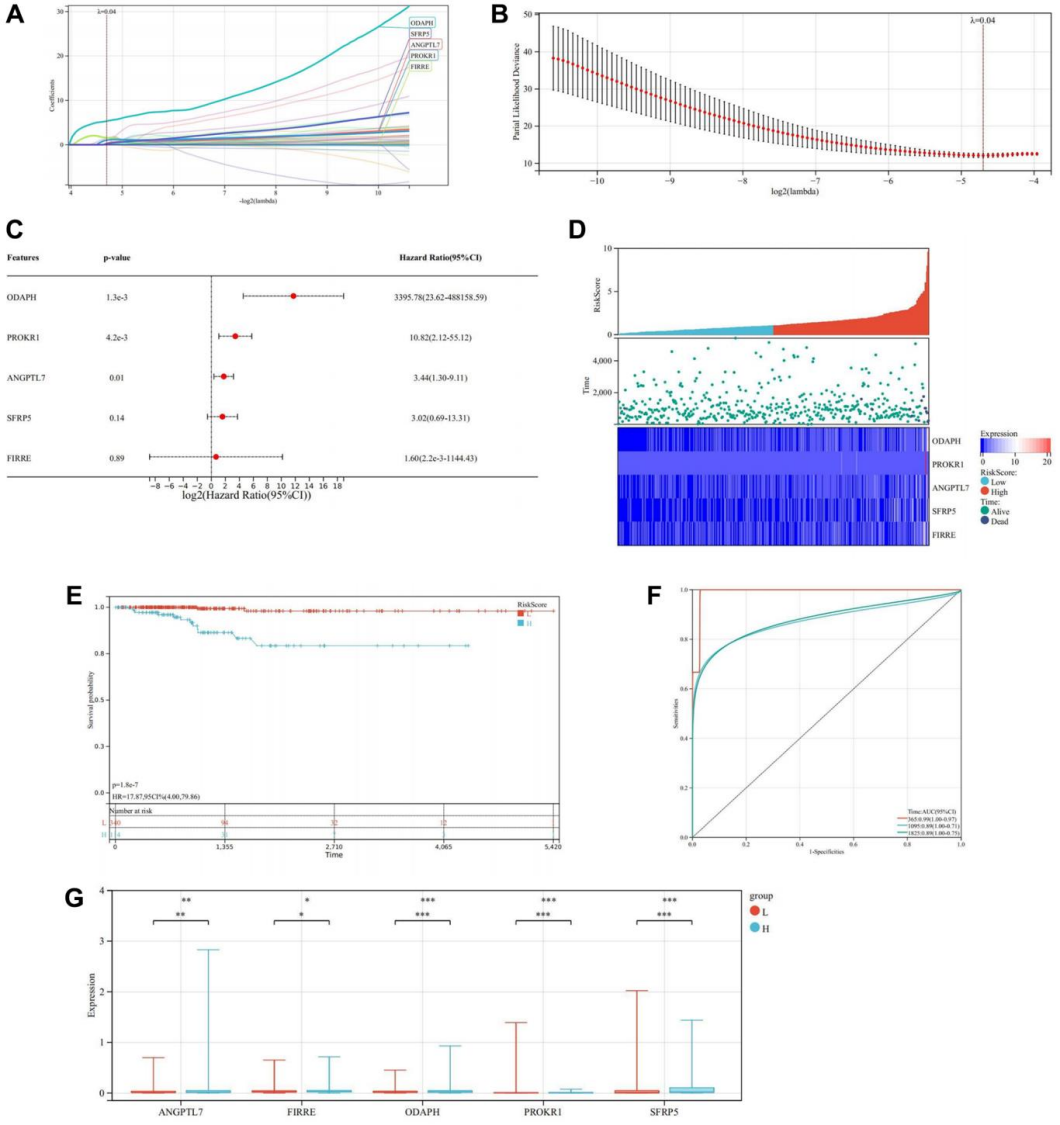
**Construction and verification of prognostic model.** (**A**) LASSO coefficient map of the prognostic genes. (**B**) After ten cross-verifications, the LASSO model of parameter selection is adjusted with minimum absolute shrinkage and selection. (**C**) Forest plot of the prognostic genes. (**D**) Heatmap of risk score. (**E**) Kaplan-Meier of high and low-risk group. (**F**) Predictive value of Cox prognostic model in THCA patients evaluated by ROC curves. (**G**) Box diagram of the prognosis genes expression in the high and low-risk groups.

LASSO regression analysis was performed to ascertain the optimal λ value through cross-validation employing 10 folds. Subsequently, five genes demonstrating significant prognostic effects were identified (Figure 12A–12D). Cox multivariate regression analysis was then employed to construct a risk model for calculating the risk score, formulated as follows: RiskScore = (0.319775943653634 × ANGPTL7) + (1.61175649349686 × FIRRE) + (5.33230222379885 × ODAPH) + (1.05543854580178 × PROKR1) + (0.196685509128214 × SFRP5). Based on the median risk score, all samples were stratified into high and low-risk groups. Kaplan-Meier survival curve analysis unveiled that the survival rate of the high-risk group was significantly lower compared to the low-risk group (Figure 12E). Furthermore, the prognostic model exhibited robust forecasting ability, as evidenced by the high AUC values for predicting the OS of THCA patients at 1, 3, and 5 years, which were 0.99, 0.89, and 0.89, respectively (Figure 12F).

The box diagram illustrated that prognosis genes ANGPTL7, FIRRE, and ODAPH were highly expressed in the high-risk group (*P* < 0.05), while PROKR1 and SFRP5 were highly expressed in the low-risk group (*P* < 0.0001) (Figure 12G).

**Figure 13:**
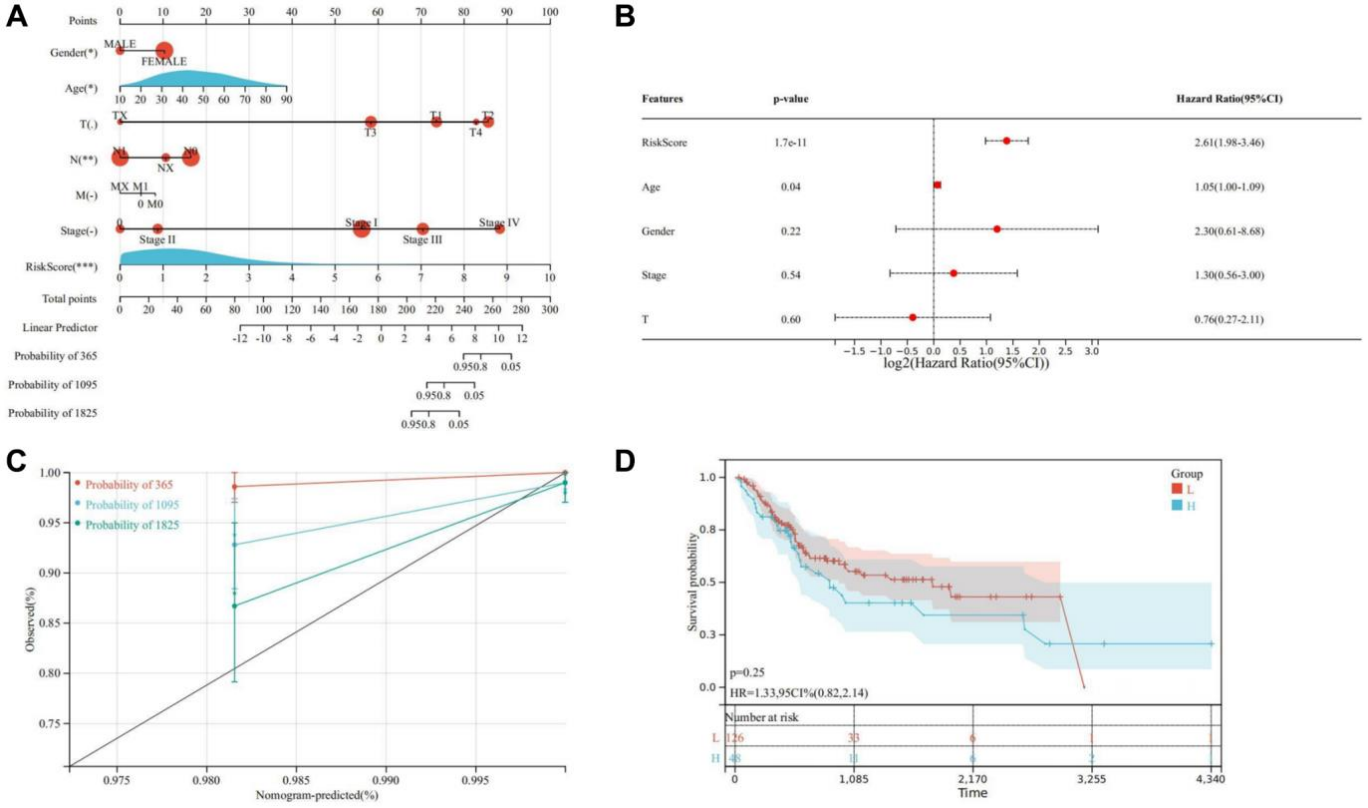
**Establishment of a nomogram model.** (**A**) Nomogram model for evaluating the prognosis of THCA patients. (**B**) Multivariate Cox regression. (**C**) Calibration curve of nomogram model. (**D**) Kaplan-Meier of the high and low-risk groups in the validation cohort. ^*^ represents *P* < 0.05, ^**^ represents *P* < 0.01, ^***^ represents *P* < 0.001, ^****^ represents *P* < 0.0001, - represents no significant difference.

Through univariate and multivariate Cox analysis, it was ascertained that age, stage, gender, and risk score served as independent prognostic factors for THCA patients. This underscores the efficacy of the prognosis model in assessing THCA risk. Additionally, a nomogram model was developed by integrating clinical factors to evaluate the prognosis of THCA patients (Figure 13A, 13B). Calibration curve analysis revealed a close fit between the calibration curve and the ideal curve, indicating high prediction accuracy of the model (Figure 13C). Similarly, consistent conclusions were obtained in the validation cohort, where significant differences in OS were observed between the high and low-risk groups, with patients in the high-risk group exhibiting poorer prognosis (Figure 13D).

**Figure 14:**
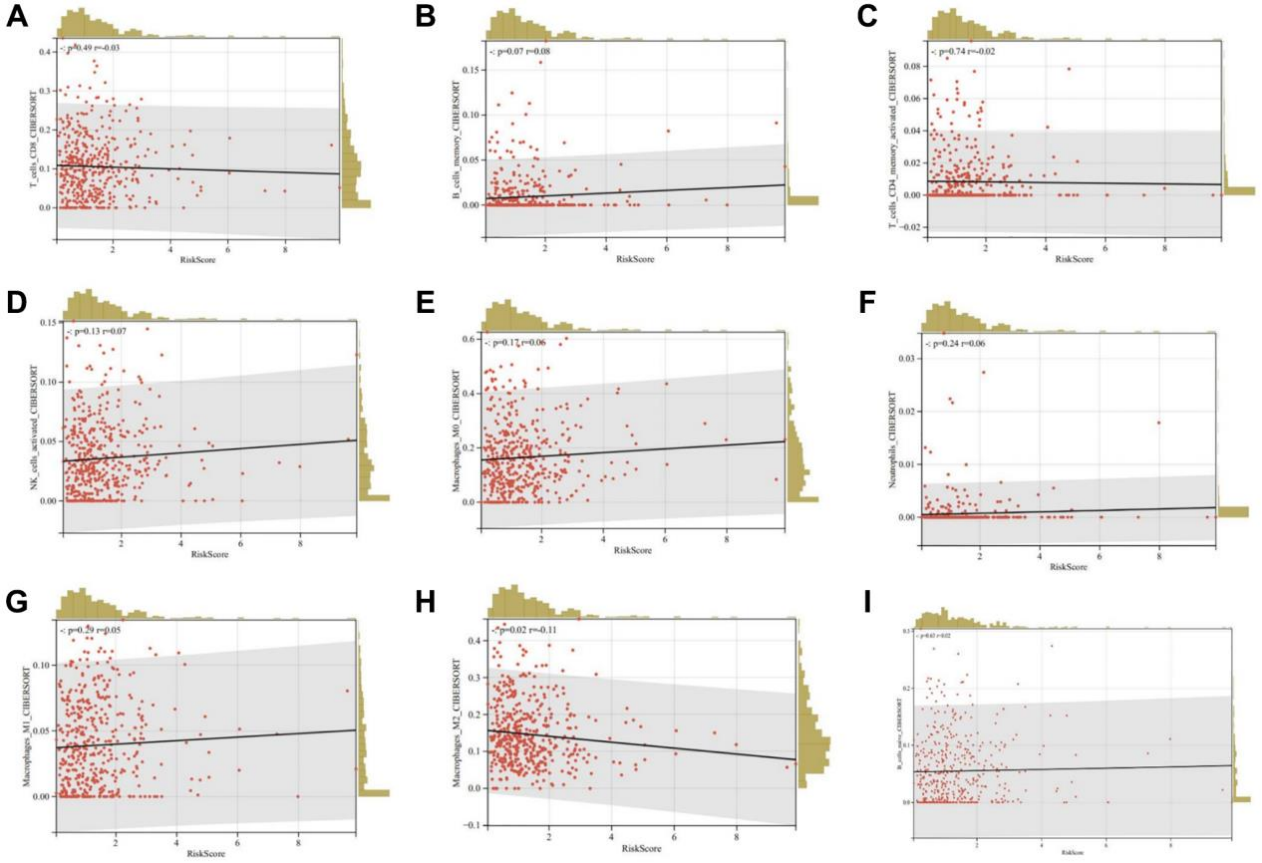
**Relationship between risk scores and immune cell infiltration.** (**A**) CD8+ T cells; (**B**) Memory B cells; (**C**) Activated memory CD4+ T cells; (**D**) Activated NK cells; (**E**) M0 macrophages; (**F**) Neutrophils; (**G**) M1 macrophages; (**H**) M2 macrophages; (**I**) Naïve B cells.

Furthermore, we evaluated the relationship between risk scores and immune cell infiltration. The results indicated that patients with increased risk scores were negatively correlated with CD8+ T cells, CD4 memory cells, and M2 macrophages (Figure 14A–14I). This suggests that patients with low-risk scores could potentially benefit more from immunotherapy.

### Verification of DEDRGs

#### Bayesian co-localization analysis

**Figure 15:**
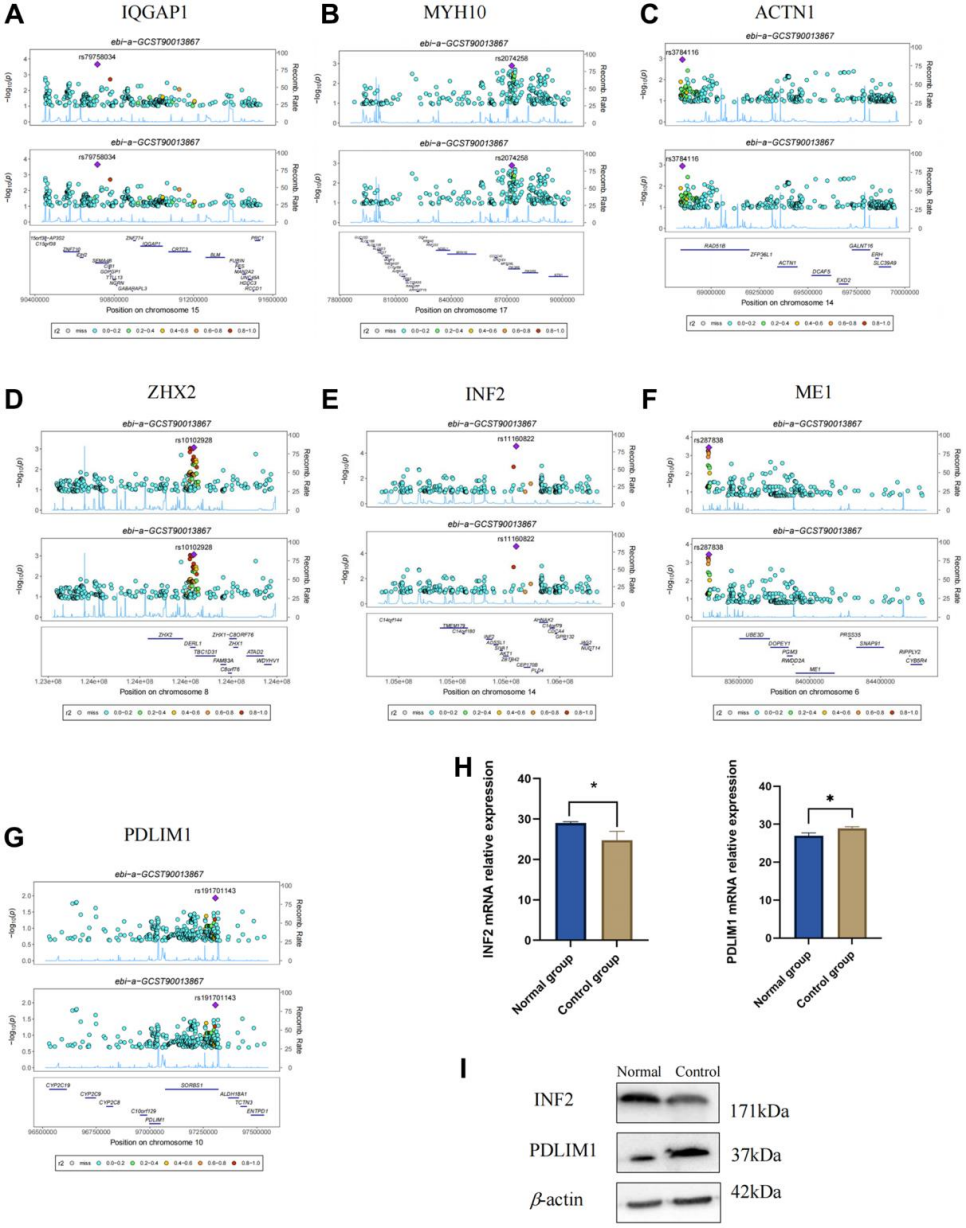
**Verification of DEDRGs by Bayesian co-localization and experiment.** (**A**–**G**) Bayesian co-localization analysis. (**H**) mRNA relative expression of PDLIM1 and INF2 (^*^P < 0.05). (**I**) Protein level of INF2 and PDLIM1 in the normal group and control group.

In the GWAS co-location analysis between DEDRGs and THCA, our investigation revealed PDLIM1 (PPH4 = 0.78) and INF2 (PPH4 = 0.99) to be collocated with THCA. Notably, our analysis did not unveil any GWAS co-location findings for FLNA with THCA (Figure 15A–15G, Supplementary Table 4). This observation suggests that the remaining five DEDRGs (IQGAP1, MYH10, ACTN1, ZHX2, ME1) do not exhibit a genetic correlation with THCA, despite their documented involvement in its pathogenesis as elucidated in our study. We postulate that observational studies may be susceptible to acquired confounding factors, such as environmental influences and acquired genetic variations.

#### Experimental verification in vitro

qRT-PCR analysis was utilized to discern the disparity in PDLIM1 and INF2 mRNA expression levels between the normal and control groups. Remarkably, a discernible decrease was observed in the expression level of INF2 mRNA within the control group (FTC-133) compared to the normal group. Conversely, the expression level of PDLIM1 mRNA within the control group (FTC-133) exhibited a marked increase relative to the normal group. These disparities yielded statistically significant results (*P* < 0.05), as depicted in Figure 15H. Western blot results indicated a significant decrease in the expression of INF2 protein in the control group, while the expression of PDLIM1 protein in the control group showed a significant increase (Figure 15I), which is consistent with the qRT-PCR validation. These results further corroborate the findings of our study.

## DISCUSSION

THCA, as an endocrine system tumor, has been associated with favorable clinical therapeutic outcomes. However, the potential for cancer cells to evade anti-tumor immune responses raises concerns regarding recurrence or metastasis [23]. Consequently, the search for novel therapeutic targets in THCA remains imperative. Currently, the precise mechanism underlying disulfidptosis in THCA remains elusive. Therefore, through an analysis of the relationship between DRGs and THCA, this study aims to identify new therapeutic targets and provide theoretical insights for the treatment of THCA.

Initially, two distinct subtypes related to DRGs were identified through consensus clustering. Cluster C1, characterized by elevated DRGs levels, exhibited a favorable prognosis, whereas cluster C2, marked by diminished DRGs levels, presented a poorer prognosis. Subsequently, eight DEDRGs (FLNA, IQGAP1, MYH10, ACTN1, ZHX2, INF2, ME1, PDLIM1) were discerned from the TCGA-THCA-DRGs dataset, with seven genes showing up-regulation and one gene displaying down-regulation. Notably, cluster C1 demonstrated heightened expression of DEDRGs, while cluster C2 exhibited diminished expression. Among these genes, FLNA, classified as an actin-binding protein (ABP), plays a pivotal role in cytoskeletal formation and is intricately involved in cellular processes such as adhesion, proliferation, migration, signal transduction, and tumorigenesis [24]. Research has elucidated that the expression of FLNA escalates in invasive breast cancer tissue concomitant with reduced differentiation [25]. Additionally, FLNA exhibits significant mutations in THCA [26]. IQGAP1, an integral player in various biological processes within the human body, plays a pivotal role in modulating cell adhesion, signal transduction, and cell division [27]. Prior investigations have implicated IQGAP1 in the invasion and metastasis of THCA [28]. Moreover, MYH10, a member of the protein-coding gene superfamily, not only participates in normal cellular physiological activities but also bears close association with cancer initiation and progression [29]. Although MYH10’s involvement in bladder cancer and lung cancer has been documented, its relationship with THCA remains unexplored. Furthermore, heightened expression of ACTN1 is intricately linked to tumor cell motility, metastasis, and invasiveness [30]. Silencing ACTN1 expression has been demonstrated to impede the proliferation of hepatocellular carcinoma (HCC) [31]. ZHX2, recognized as a tumor suppressor gene, orchestrates lipid metabolism regulation, suppresses cell proliferation [32], and influences the immune microenvironment [33]. Notably, ZHX2 has been implicated in the occurrence of brain metastasis in THCA [34]. INF2, through its involvement in mitochondrial division, exerts effects on the proliferation and invasion of numerous tumors [35]. Furthermore, INF2 has been shown to inhibit THCA proliferation via the Hippo pathway [36]. ME1 primarily participates in lipid metabolism and tumor progression [37]. Although ME1 serves as a transcriptional target of thyroxine, its association with THCA remains unexplored [38]. PDLIM1, a cytoskeleton protein, interacts with actin stress fibers [39]. Studies have revealed diverse functions of PDLIM1 across various tumor tissues, wherein it can either impede epithelial-mesenchymal transition (EMT) and tumor cell infiltration and metastasis or promote tumor development [40]. Notably, PDLIM1 up-regulation has been observed in THCA [41]. Furthermore, we corroborated the findings related to DEDRGs in the GEPIA and HPA databases, which aligned with the existing literature. Additionally, analysis from the TIMER database revealed a robust correlation between DEDRGs and immune cells. Collectively, these results underscore the close association of DEDRGs with THCA and immune infiltration [23].

In our subsequent analysis, we employed functional enrichment analysis to delve into the potential biological functions and molecular mechanisms orchestrated by DEDRGs. The findings from this endeavor unveiled remarkable enrichments in pivotal pathways, including the MAPK, PPAR signaling pathway, and Proteoglycans in cancer. Among these pathways, the MAPK signaling pathway emerged as a central conduit bridging cell membrane receptors with nuclear events, playing a pivotal role in initiating and propelling various malignancies [42]. Notably, the strikingly elevated BRAF mutation rate, peaking at 76.2% in THCA, underscores its paramount importance in driving the progression of THCA by activating the MAPK signaling pathway [43]. Furthermore, the regulation mediated by PPAR, influencing thyroid peroxidase, thyroglobulin, and thyrotropin receptor gene promoters, facilitates the intricate process of thyroid cell differentiation [44]. These insights shed light on the intricate interplay between signaling pathways and genetic alterations, offering valuable perspectives into the molecular landscape of THCA pathogenesis [24–41].

The tumor microenvironment has garnered recognition as a crucial determinant influencing tumor initiation, progression, treatment response, and prognosis [45]. However, the intricate relationship between genes associated with the tumor microenvironment and THCA remains partially understood. Therefore, we embarked on a comparative analysis of immune cell infiltration between two distinct subtypes. Our findings unveiled that cluster C1, characterized by heightened DRGs levels, exhibited elevated immune cell infiltration, while cluster C2 demonstrated higher immune, estimate, and stromal scores. This led us to hypothesize that cluster C2 signifies an immune rejection type, whereas cluster C1 represents an immune inflammation type. Remarkably, immune checkpoint molecules TIGIT, PD-L1, CTLA4, and CD274 displayed heightened expression in cluster C1. Hence, we conjectured that DEDRGs might modulate disulfidptosis and reshape the immune microenvironment, potentially offering therapeutic targets for THCA.

Following the insightful Bayesian co-localization analysis, PDLIM1 and INF2 emerged as candidates collocated with THCA. However, the genetic correlation between THCA and the remaining five DEDRGs (IQGAP1, MYH10, ACTN1, ZHX2, ME1) did not find substantiation, possibly due to susceptibility to acquired confounding factors. Based on these compelling findings, we selected PDLIM1 and INF2 for experimental validation. Our results revealed that both the mRNA and protein expression levels of INF2 in the control group (FTC-133) were notably lower than those in the normal group, whereas the mRNA and protein expression levels of PDLIM1 in the control group (FTC-133) were markedly higher than those in the normal group (*P* < 0.05). These experimental validations provide robust confirmation of our study’s hypotheses [42–44].

The establishment of prognostic models represents a pivotal stride in effectively predicting the outcomes of tumor patients [46, 47]. In the realm of molecular characteristics, specialized models like the m1Ascore have been devised to assess individual patients’ m1A modification patterns [48]. Remarkably, our study pioneers a comprehensive analysis delving into potential therapeutic targets, mechanisms, and candidate therapeutic agents for THCA, with a focused exploration into disulfidptosis and the immune microenvironment. Leveraging bioinformatics methodologies, we forecasted that interventions targeting DEDRGs, prognostic genes, and pathways such as MAPK and PPAR could hold transformative potential in THCA treatment by modulating disulfidptosis and the immune microenvironment. In comparison with prior prognostic features, our study’s prognostic model demonstrates a heightened AUC level in the verification set, signifying enhanced predictive accuracy. Moreover, the validation through Bayesian co-localization analysis and *in vivo* experiments further bolsters the reliability and precision of our findings.

Nevertheless, it is imperative to acknowledge several inherent limitations in our study. Primarily, all our findings stem from publicly available databases, and our experimental validations were exclusively confined to *in vitro* settings. Thus, the imperative need for further *in vivo* experiments and additional functional assays to robustly authenticate our results cannot be understated. Secondly, the intricate mechanistic interplay between disulfidoptosis and the tumor microenvironment warrants deeper exploration and elucidation. Sustained research endeavors in these domains will be pivotal in augmenting our comprehension and propelling therapeutic strategies forward for THCA. Thirdly, the construction of a prognostic risk model for THCA based on immune correlation holds significant promise. Such a model could offer comprehensive insights into the survival rate and response to immunotherapy among THCA patients, underscoring the importance of future investigations in this direction [45–48].

## CONCLUSION

In our investigation, we adeptly delineated two distinct subtypes intricately associated with DRGs, thereby illuminating their unique infiltration patterns and divergent survival prognosis. This delineation provides crucial insights into the complex interplay between DRGs and THCA progression. Moreover, our findings underscore the potential regulatory role of DEDRGs in shaping the tumor immune microenvironment, notably through modulation of key signaling pathways such as MAPK and PPAR. These revelations not only deepen our understanding of THCA pathogenesis but also furnish a solid theoretical foundation for the development of innovative therapeutic strategies aimed at combatting this disease.

## Supplementary Materials

Supplementary Figures

Supplementary Tables 1-3

Supplementary Table 4
