# Supplementary material for: Identification of disulfidptosis-associated genes and characterization of immune cell infiltration in thyroid carcinoma
Source: Aging (Albany NY). 2024 Jun 4;16(11):9753–83. doi: 10.18632/aging.205897 (PMC11210228; doi:10.18632/aging.205897)
Supplement: Supplementary Figures [file aging-16-205897-s001.pdf]

SUPPLEMENTARY FIGURES

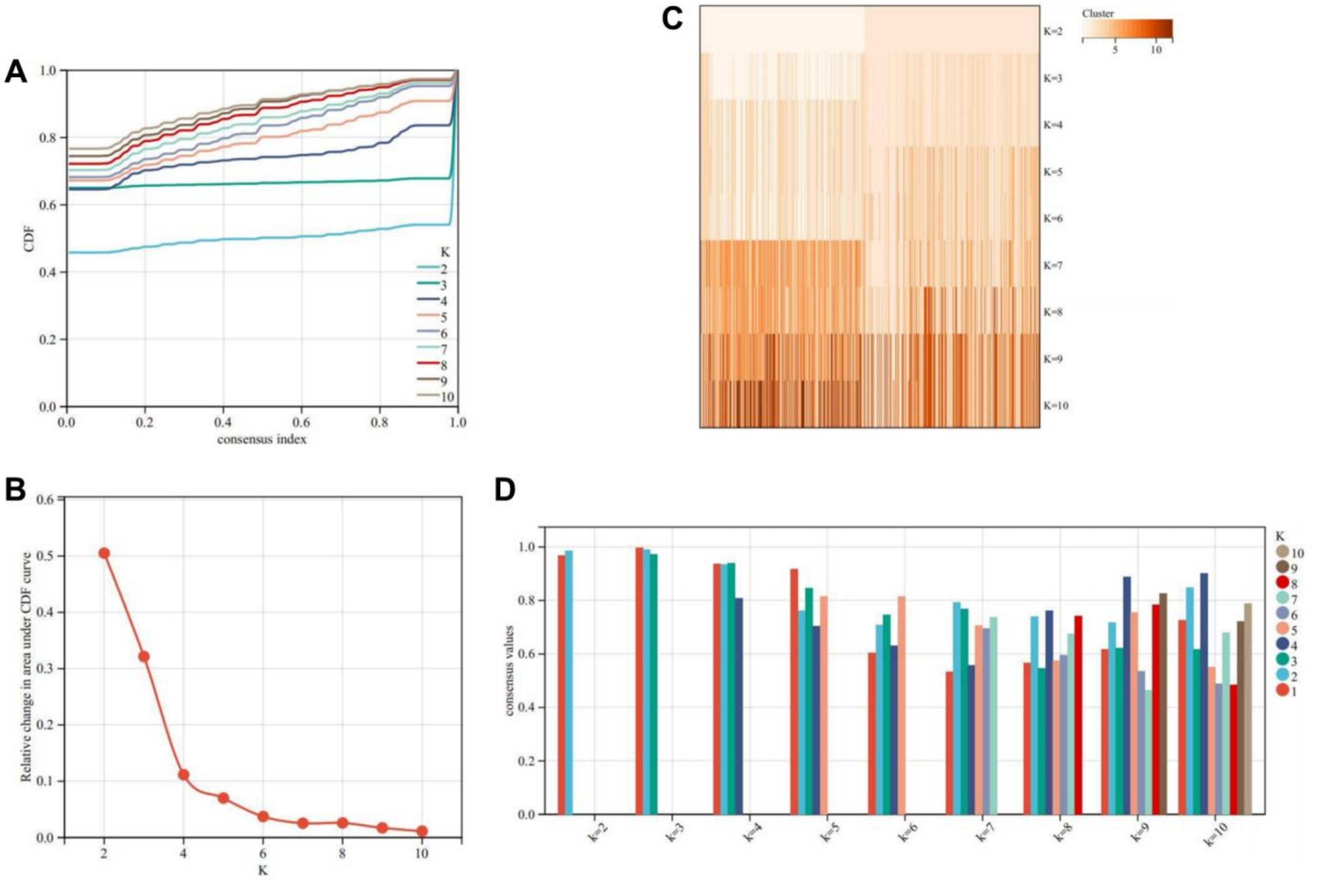

Supplementary Figure 1 Consensus clustering analysis. (**A**) Represents the CDF curve when k = 2−10. (**B**) Represents the delta area fraction of the CDF curve when k = 2–10. (**C**) Represents the tracking chart when k = 2–10. (**D**) Represents the consensus values when k = 2–10.

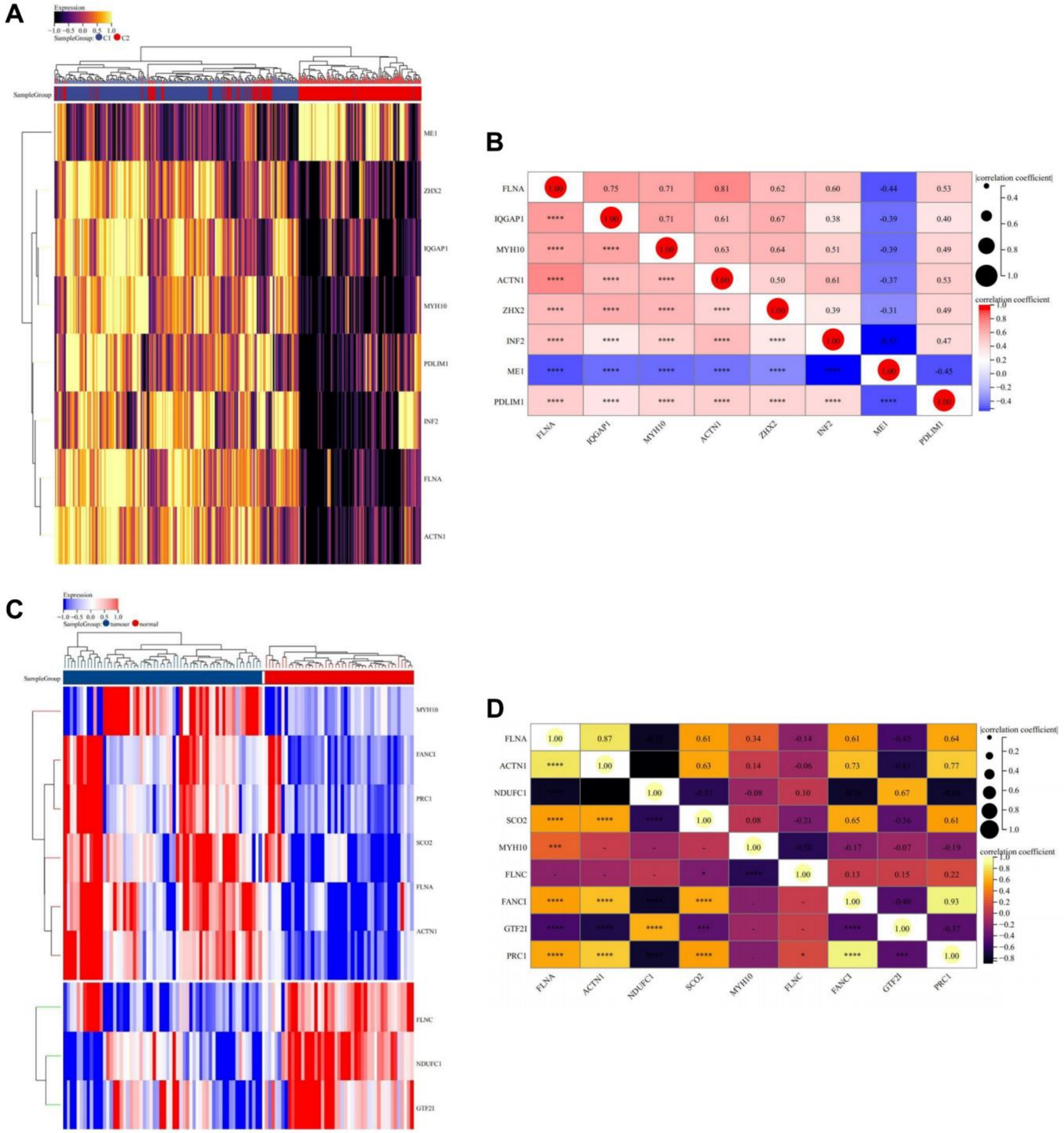

Supplementary Figure 2. Differential expression and correlation analysis. (**A**) Heatmap of DEDRGs in cluster C1 and C2 subtypes. (**B**) Correlation analysis of DEDRGs in TCGA-THCA. (**C**) Heatmap of DEDRGs in GSE33630. (**D**) Correlation analysis of DEDRGs in GSE33630. * represents $P < 0.05$, *** represents $P < 0.001$, **** represents $P < 0.0001$, - represents no significant difference.

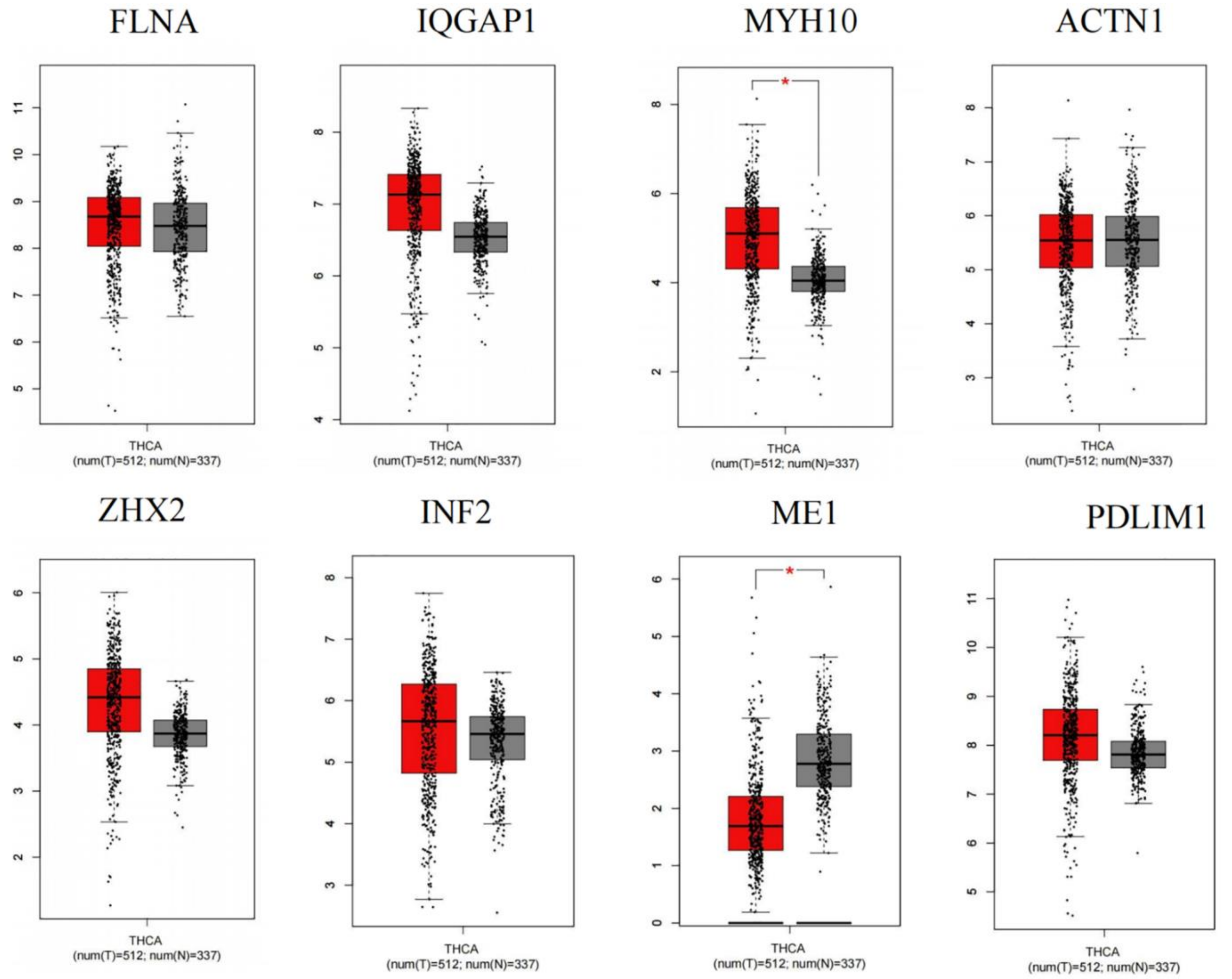

Supplementary Figure 3. Expression difference of the DEDRGs in the GEPIA database. Red represents tumor samples and gray represents normal tissues.

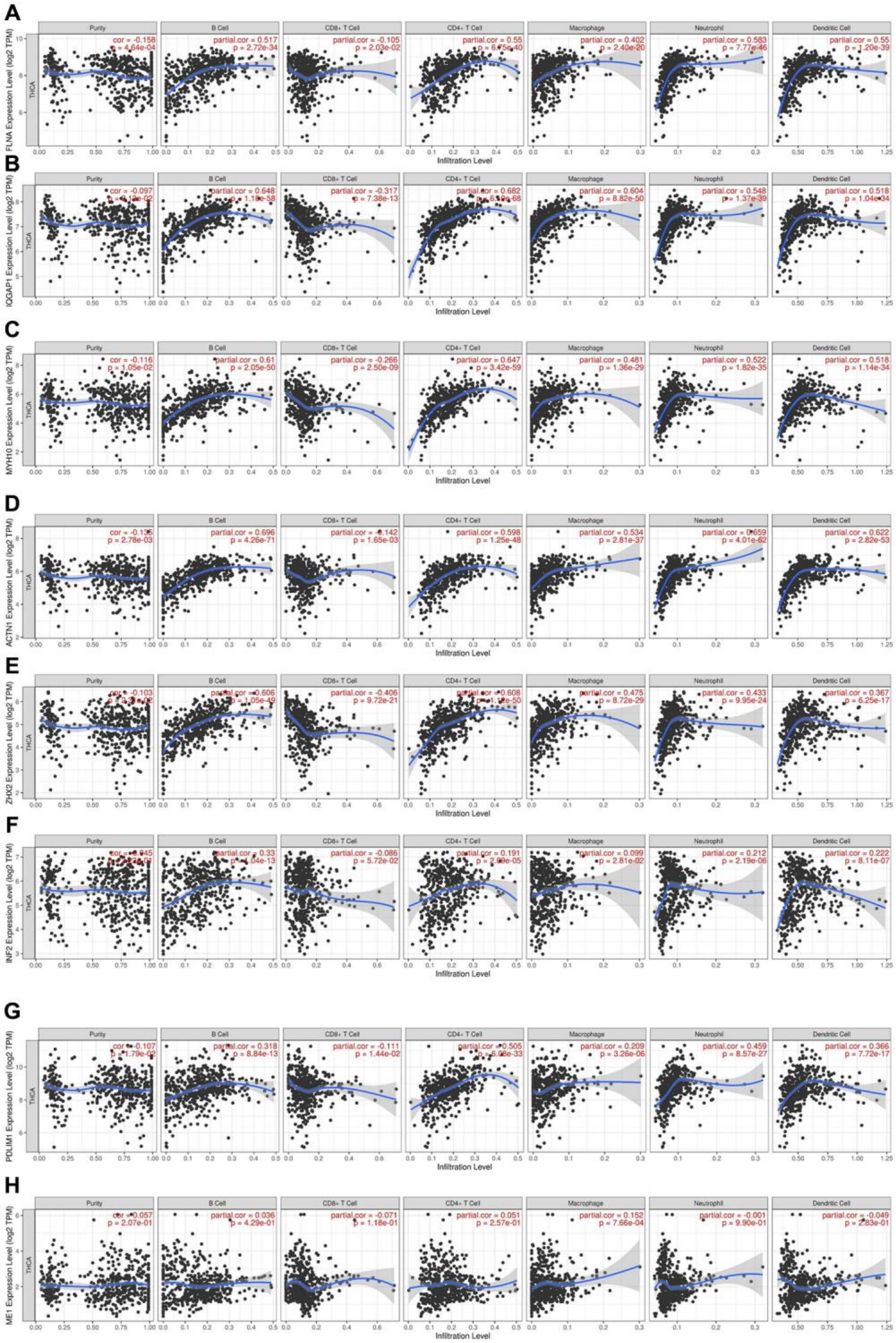

Supplementary Figure 4. Relationship between the DEDRGs and immune cell infiltration in the TIMER database. (**A**) FLNA; (**B**) IQGAP1; (**C**) MYH10; (**D**) ACTN1; (**E**) ZHX2; (**F**) INF2; (**G**) PDLIM1; (**H**) ME1.
