# Supplementary material for: Identification of disulfidptosis-associated genes and characterization of immune cell infiltration in thyroid carcinoma
Source: Aging (Albany NY). 2024 Jun 4;16(11):9753–83. doi: 10.18632/aging.205897 (PMC11210228; doi:10.18632/aging.205897)
Supplement: Supplementary Tables 1-3 [file aging-16-205897-s002.pdf]

SUPPLEMENTARY TABLES

Supplementary Table 1. Characteristic of the THCA patients in TCGA database.

	TCGA
No. of patients	507
Age (%)	
≤45	268 (52.9)
>45	139 (47.1)
Gender (%)	
Female	371 (73.2)
Male	136 (26.8)
Stage (%)	
I	285 (56.2)
II	52 (10.2)
III	113 (22.4)
IV	55 (10.8)
NA	2 (0.4)
TNM (%)	
T	
T1	144 (28.4)
T2	167 (33)
T3	171 (33.7)
T4	23 (4.5)
TX	2 (0.4)
N	
N0	231 (45.5)
N1	226 (44.6)
NX	50 (9.9)
M	
M0	283 (55.9)
M1	9 (1.79)
MX	214 (43.2)
NA	1 (0.01)
Survival status	
Median OS (day)	944
Ending (%)	
Survival	491 (96.8)
Death	16 (3.2)

Abbreviations: T: primary tumor; N: regional lymph nodes; M: distant metastasis; NA not available.

Supplementary Table 2. Disulfidptosis-related genes (DRGs) in the study.

Gene names
AAAS
ACTB
ACTN1
ACTN2
ACTN3
ACTN4
ANP32E
ARMC6
ARNT
ATG5
ATXN10
BOP1
CAPZB
CD2AP
CHCHD3
CHD4
CNOT1
DBN1
DHX9
DSTN
EBLN2
EPAS1
FANCI
FLNA
FLNB
FLNC
GCN1L1
GLUD1
GTF2I
GYS1
HNRNPA3
HNRNPH1
HNRNPH2
HNRNPH3
HNRNPM
HNRNPU
INF2
IPO4
IPO7
IQGAP1
LGALS13
LRPPRC
ME1
MRPS17
MYH1
MYH10
MYH11
MYH13
MYH14
MYH2
MYH3
MYH4
MYH6
MYH7

MYH7B
MYH8
MYH9
MYL6
MYL6B
NCKAP1
NDUFA10
NDUFA11
NDUFB10
NDUFB11
NDUFB6
NDUFC1
NDUFS1
NDUFS2
NIPBL
NLN
NSUN2
NUBPL
OXSM
PCBP1
PCBP2
PCBP3
PDLIM1
PML
PPIH
PPM1F
PRC1
PRDX1
RNH1
RPA1
RPN1
RRP1
RUFY1
SAFB
SAFB2
SAMM50
SART3
SCO2
SLC3A2
SLC7A11
SMPD4
SPG20
SQSTM1
STRAP
TARDBP
TDP43
TLN1
TLN2
TNKS1BP1
TRIP6
UBASH3B
ZHX2

Supplementary Table 3. Real-time polymerase chain reaction primers.

Gene	Sequence (5′–3′)
PDLIM1-F	CTCCAACCTACTAATTCCGACCTTCAC
PDLIM1-R	ACGCACTCAACGGCTTATCACAC
INF2-F	CTCCAACCTACTAATTCCGACCTTCAC
INF2-R	ACGCACTCAACGGCTTATCACAC
